# Heritable components of the human fecal microbiome are associated with visceral fat

**DOI:** 10.1186/s13059-016-1052-7

**Published:** 2016-09-26

**Authors:** Michelle Beaumont, Julia K. Goodrich, Matthew A. Jackson, Idil Yet, Emily R. Davenport, Sara Vieira-Silva, Justine Debelius, Tess Pallister, Massimo Mangino, Jeroen Raes, Rob Knight, Andrew G. Clark, Ruth E. Ley, Tim D. Spector, Jordana T. Bell

**Affiliations:** 1 Department of Twin Research & Genetic Epidemiology, King’s College London, St Thomas’ Hospital, 3rd Floor, South Wing, Block D, London, SE1 7EH UK; 2 Department of Microbiology, Cornell University, Ithaca, NY 14853 USA; 3 Department of Molecular Biology and Genetics, Cornell University, Ithaca, NY 14853 USA; 4 Department of Chemistry and Biochemistry, University of Colorado, Boulder, CO 80309 USA; 5 Biofrontiers Institute, University of Colorado, Boulder, CO 80309 USA; 6 Howard Hughes Medical Institute, Boulder, CO 80309 USA; 7 Present address: Departments of Pediatrics and Computer Science and Engineering, University of California San Diego, La Jolla, CA 92093 USA; 8 Department of Microbiome Science, Max Planck Institute for Developmental Biology, Tübingen, Germany; 9 Department of Microbiology and Immunology, KU Leuven – University of Leuven, Leuven, Belgium; 10 VIB lab for Bioinformatics and (eco-)systems biology, Leuven, Belgium

**Keywords:** Fecal microbiome, Obesity, Visceral fat, Heritability, Genetic association, Twins

## Abstract

**Background:**

Variation in the human fecal microbiota has previously been associated with body mass index (BMI). Although obesity is a global health burden, the accumulation of abdominal visceral fat is the specific cardio-metabolic disease risk factor. Here, we explore links between the fecal microbiota and abdominal adiposity using body composition as measured by dual-energy X-ray absorptiometry in a large sample of twins from the TwinsUK cohort, comparing fecal 16S rRNA diversity profiles with six adiposity measures.

**Results:**

We profile six adiposity measures in 3666 twins and estimate their heritability, finding novel evidence for strong genetic effects underlying visceral fat and android/gynoid ratio. We confirm the association of lower diversity of the fecal microbiome with obesity and adiposity measures, and then compare the association between fecal microbial composition and the adiposity phenotypes in a discovery subsample of twins. We identify associations between the relative abundances of fecal microbial operational taxonomic units (OTUs) and abdominal adiposity measures. Most of these results involve visceral fat associations, with the strongest associations between visceral fat and *Oscillospira* members. Using BMI as a surrogate phenotype, we pursue replication in independent samples from three population-based cohorts including American Gut, Flemish Gut Flora Project and the extended TwinsUK cohort. Meta-analyses across the replication samples indicate that 8 OTUs replicate at a stringent threshold across all cohorts, while 49 OTUs achieve nominal significance in at least one replication sample. Heritability analysis of the adiposity-associated microbial OTUs prompted us to assess host genetic-microbe interactions at obesity-associated human candidate loci. We observe significant associations of adiposity-OTU abundances with host genetic variants in the *FHIT*, *TDRG1* and *ELAVL4* genes, suggesting a potential role for host genes to mediate the link between the fecal microbiome and obesity.

**Conclusions:**

Our results provide novel insights into the role of the fecal microbiota in cardio-metabolic disease with clear potential for prevention and novel therapies.

**Electronic supplementary material:**

The online version of this article (doi:10.1186/s13059-016-1052-7) contains supplementary material, which is available to authorized users.

## Background

Obesity has rapidly become a global public health problem, with obesity-related disease now one of the leading causes of preventable death worldwide [1]. Although overall obesity poses a global health epidemic, it is the accumulation of excess abdominal fat that is a critical risk factor for cardiovascular and metabolic disease [2]. Changes in diet and a sedentary lifestyle can partly explain the rise in obesity, and family and twin studies also show a genetic influence, with obesity heritability estimates of 0.60–0.70 [3–6]. Genome-wide association studies (GWASs) have identified genetic risk factors [7–9], but genetic variants detected to date explain less than 3 % of the heritability of obesity, with a prediction ability of up to 20 %, suggesting a role for other mechanisms [10].

Recent insights show that the gut microbiota may play a crucial role in obesity and cardio-metabolic disease risk. Many studies have linked different aspects of the fecal microbiome to obesity [11–17]. However, in most cases the causal mechanisms leading to these associations are unclear, although several theories have been suggested, including alterations in energy harvest from food [18] and an increase in potential inflammatory microbes [19]. There are also inconsistencies in the taxa associated with obesity [16] that may be explained in part by study design differences such as control of diet and sequencing platforms, but could also be due to differences in collective bacterial gene function rather than the species community composition [20]. Another complicating factor that varies among studies is the quantification of obesity. While most human studies consider body mass index (BMI) as the measure of obesity [11, 21, 22], mouse studies typically use epididymal fat weight [23] or dual-energy X-ray absorptiometry (DXA)-derived measures of total body fat [18, 24]. BMI is an imprecise measure of adiposity and measures overall mass without distinction between lean and fat mass [25]. Estimates of visceral fat, however, have stronger associations with obesity-related cardio-metabolic diseases, such as type 2 diabetes and cardiovascular disease [26–28], but have typically been difficult to measure in humans and have yet to be linked with variation in the human fecal microbiome.

Previous studies have attempted to tackle heritability of attributes of the microbiome. Zoetendal et al. [29] found that monozygotic (MZ) twins had more similar microbiomes than marital partners or unrelated individuals, suggesting either a role for host genotype in gut microbiome colonisation or mother-to-child transmission of microbes. In addition, a study of *Methanobrevibacter* carriage concordance rate in twins showed higher concordance in MZ twins [30]. A recent study by Goodrich et al. [31] was the first large-scale analysis to report heritability of the human fecal microbiota, with the relative abundance of 16S rRNA gene sequences belonging to the family *Christensenellaceae* showing the most variance attributed to host genetic effects. *Christensenellaceae* was also enriched in abundance in the microbiomes of low-BMI individuals. Here, we build upon these findings to explore the association between the human fecal microbiome and abdominal adiposity as the main risk factor for cardio-metabolic disease risk. We obtained DXA-based measures of abdominal adiposity, specifically, visceral fat mass, subcutaneous fat mass and previously reported trunk fat measures [32], as well as body fat distribution, in a larger dataset of twins, including a subset of the twin sample profiled by Goodrich et al. [33] and a subset of the twin sample profiled by Jackson et al. [34]. We show that heritable components of the human fecal microbiome [31, 33] are significantly associated with visceral fat, confirming the key role of the microbiome in cardio-metabolic disease risk. We further identify a link between fecal microbiome profiles, visceral fat and subcutaneous fat with genetic variants in obesity candidate genes, providing potential insights into mechanisms to relate the fecal microbiome to cardio-metabolic disease risk.

## Results

Measures of adiposity were obtained from an unselected sample of 3666 predominantly female twins from the TwinsUK cohort (TUK-D), which included 1044 MZ and 789 dizygotic (DZ) twin pairs (average age 63 years (range 32–87); 96.4 % female). Fecal microbiome profiles were available for 1313 of these individuals (496 MZ, 594 DZ and 223 unrelated individuals; average age 63 years (range 32–87); 96.4 % female). The demographics for these samples can be found in Table 1 and Additional file 1: Table S7. Fecal microbiome profiles included 601 previously published profiles from Goodrich et al. [33], an additional 671 profiles recently reported within Jackson et al. [34] and 41 additional twin profiles [33]. All fecal samples underwent 16S rRNA profiling (V4 region) gene sequencing on the Illumina MiSeq platform, providing 2135 operational taxonomic units (OTUs) at 97 % sequence identity.

**Table 1:** Description of the TwinsUK discovery sample

	Full dataset^a^	Microbiome subset^b^
No. of samples	3666	1313
Sex		
Female	3296	1266
Male	370	47
Zygosity		
MZ	2088	496
DZ	1578	594
Unrelated	0	223
Age (mean (range))	63 (32–87)	63 (32–87)
BMI (mean (range))	26.1 (15.7–49.9)	26.1 (16.2–45.9)
Ethnicity		
European	3511	1295
Other	102	9
Unknown	53	8

^a^Summaries are shown for both the extended dataset of 3666 twins used in the phenotype heritability analyses and for the microbiome sample subset of 1313 individuals. ^b^Additional file 1: Table S7 includes extended descriptions of the microbiome data subset

### Adiposity and visceral fat heritability

**Fig. 1:**
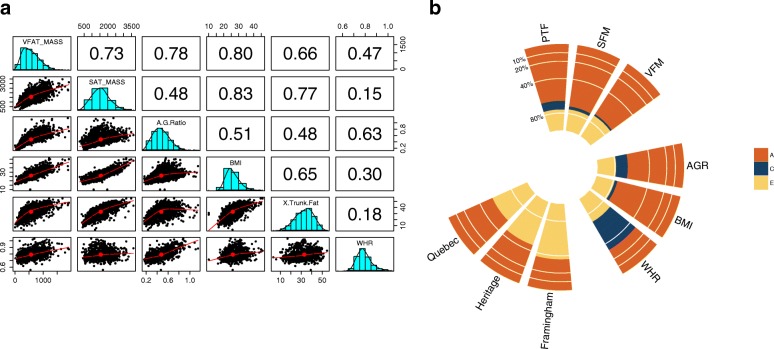
Distribution and heritability of adiposity phenotypes. **a** Scatterplot matrix showing the distribution and correlation between six adiposity measures in 3666 twins. The distribution of each phenotype (prior to normalisation) is shown along the diagonal. The *lower panel* shows scatterplots for each pair of adiposity phenotypes, and the *upper panel* denotes the coefficients of determination. **b** Heritability of six adiposity measures in the TwinsUK cohort, as well as visceral fat measures in three independent cohorts: Framingham [39], Quebec [41] and Heritage [40]. The total variance of each adiposity phenotype is decomposed into variance components attributed to additive genetics (A) or narrow-sense heritability (*h* ^2^), common environment (C) and unique environment (E)

We studied six adiposity measures in total, and these included three measures of abdominal adiposity (visceral fat mass (VFM), subcutaneous fat mass (SFM), percentage trunk fat (pTF)), two measures of body fat distribution (android/gynoid ratio (AGR) and waist/hip ratio (WHR)) and one measure of overall obesity, BMI. Adiposity was estimated using DXA-derived measures, which have been shown to be reliable alternatives [35–38] to traditional computed tomography (CT) and magnetic resonance imaging scan-based measures of adiposity. The majority of these adiposity measures have been previously explored in the TwinsUK cohort; however, VFM and AGR are newly obtained phenotypes. The new measure of VFM was highly correlated with other abdominal and overall adiposity measures, including BMI (Fig. 1a). Twin-based heritability analysis of VFM showed evidence of a significant additive genetic component, or heritability (*h*^2^), contributing to 0.70 (95 % CI = 0.58–0.74) of the total variance in VFM. The VFM heritability estimate remained high after adjustment for BMI (0.64, see Additional file 2). We obtained comparable estimates for the heritability of SFM (*h*^2^ = 0.72 (95 % CI = 0.60–0.77)), pTF (*h*^2^ = 0.66 (95 % CI = 0.55–0.77), AGR (*h*^2^ = 0.65 (95 % CI = 0.55–0.76)), BMI (*h*^2^ = 0.75 (95 % CI = 0.68–0.80) and a slightly lower estimate for WHR (*h*^2^ = 0.32 (95 % CI = 0.24–0.40)), in line with previous studies [39–41] (Fig. 1b).

### The twin fecal microbiome and its heritability

The human fecal microbiome in the dataset of 1313 twins comprised Firmicutes as the most dominant phylum (51 %), followed by Bacteroidetes (39 %) and Proteobacteria (4 %). These estimates are comparable to previously published results [33] and reflect a typical Western fecal microbiome. Using twins, we then explored evidence for heritability in the gut microbial profiles (relative abundances of OTUs), extending the results of Goodrich et al. [33] in the larger sample of 1313 twins using the same methods for OTU heritability. Altogether, OTU heritability in this dataset ranged between 0 and 0.42, and the average estimate over all OTUs was 0.07. The most heritable microbe was an OTU classified as *Clostridium perfringens* (*h*^2^ = 0.42 (95 % CI 0.23–0.51)) (Additional file 1: Table S1). The family *Christensenellaceae* was the most heritable family reported in Goodrich et al. [33], and while OTUs representing *Christensenella* were not the top ranked in this larger dataset, *Christensenella* OTU heritability remained high with one OTU, Greengenes OTU 176318, showing a heritability of 0.31 (95 % CI 0.21–0.41). The microbial heritability estimates presented here are overall consistent with the original microbial twin-based heritability findings from Goodrich et al. [31] and with recent extended heritability estimates from the extended TwinsUK cohort [33]. For example, at the genus level, heritability estimates across studies show a correlation of 0.67 between this TUK-D dataset and the results in Goodrich et al. [31] (Additional file 3: Figure A) and 0.77 between TUK-D and Goodrich et al. [33] (Additional file 3: Figure B). Approximately 6 % of the dataset (122 OTUs) had evidence for at least moderate heritability (*h*^2^ > 0.2), and these were present in at least 25 % of individuals (Additional file 1: Table S1).

### Fecal microbiome diversity is strongly linked to obesity and central adiposity

**Fig. 2:**
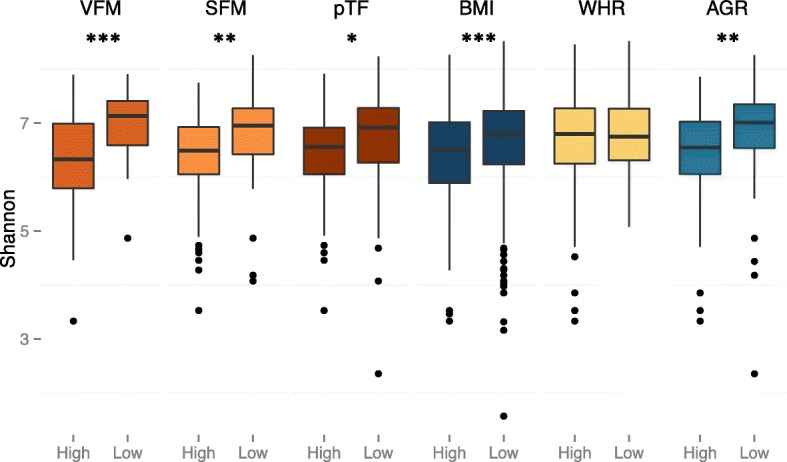
Alpha diversity of the fecal microbiome in individuals with high and low fat content. For each phenotype, individuals who were more than 1.5 standard deviations from the mean of the phenotype were assigned to high and low phenotype groups respectively. Alpha diversity measures (using Shannon diversity) were compared between the high and low phenotype groups (Wilcoxon test * = 0.05 ** = 0.001 *** = 0.0001)

Microbial diversity (alpha diversity) in obese individuals has been reported to be lower than that of lean individuals [15, 31]. Here we compared estimates of Shannon diversity for the subjects’ fecal microbiomes with all adiposity measures using a linear mixed effects model, adjusting for diet (see Methods), age, sex and family relatedness. As in previous reports [15, 31, 42], we observed a significant negative association between Shannon diversity and all adiposity phenotypes (Fig. 2, Additional file 4). VFM showed the most significant association with alpha diversity (beta = −0.14, se = 0.27, *P* = 4.13 × 10^−7^) and WHR showed the least significant association (beta = −0.05, se = 0.031, *P* = 0.097). All measures but WHR were significantly associated with diversity; therefore, alpha diversity measures in our sample are not only negatively associated with obesity but are also significantly lower in individuals with greater abdominal adiposity and visceral fat.

### Fecal microbiome profiles associate with central adiposity across twins

**Fig. 3:**
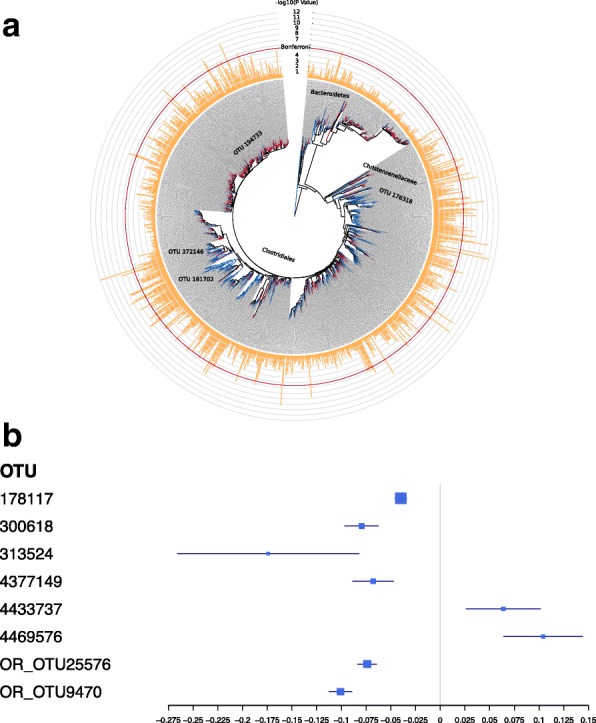
Associations between fecal microbiome 16S OTUs and visceral fat in the TwinsUK and replication datasets. **a** The *inner circle* denotes the phylogenetic tree of OTUs, produced using iTOL [93] based on Greengenes May 2013 tree filtered for the OTUs in the sample. Tree leaves are coloured according to the direction of association with visceral fat, where *blue* indicates a negative association, while *red* indicates a positive association. The *outer circle* denotes the significance of each OTU-visceral fat association, where *P* values are plotted as –log_10_ (*P* value), and the *red line* shows the Bonferroni significance threshold. The figure highlights the most-associated OTU in the sample (OTU 372146), as well as the two closed-reference OTUs that were significantly associated with host genetic variants in genes *FHIT* (OTU 181702) and *ELAVL4* (194733). It also highlights the heritable *Christensenellaceae* OTU 176318. The figure also denotes the tree branches containing members of *Clostridiales*, *Bacteroides* and *Christensenellaceae* to accompany results and discussion in the main text. **b** Forest plot of beta coefficients with confidence intervals of eight OTUs that replicated robustly in a meta-analysis of three independent cohorts (TUK-R, AG and FGFP)

We investigated the association of each OTU with all adiposity traits, including BMI, across individuals. Of the approximately 12,000 OTU-phenotype associations considered, 3217 were nominally significant, and 149 OTU results surpassed the Bonferroni correction (*P* = 3.90 × 10^−6^). The 149 significant microbial-adiposity associations involved 97 unique OTUs (Additional file 1: Table S2), and these fell within either the Firmicutes or Bacteroidetes phylum, and most within the *Ruminococcaceae* family. Visceral fat (VFM) associations made up the highest proportion of significant results surpassing the Bonferroni threshold (45 %, Fig. 3a). The peak result was an OTU classified as *Oscillospira* (Greengenes OTU 372146), which was associated with VFM (*P* = 1.93 × 10^−12^). *Ruminococcaceae* OTUs featured prominently in the top significant results, along with a number of other OTUs within the *Lachnospiraceae* family. Given the importance of VFM and AGR in cardiovascular risk, we were interested in determining potential microbial markers of cardiovascular risk. OTUs within *Oscillospira*, *Lachnospira* and *Ruminococcus* all showed negative associations with VFM and AGR, suggesting a potential protective role for these bacteria in cardiovascular risk (Fig. 3, Additional file 1: Table S2). *Blautia* OTUs showed a positive association with VFM and AGR and may be a microbial marker candidate for cardiovascular risk (Fig. 3a, Additional file 1: Table S2). These results support the crucial role of the microbiome towards visceral fat as a marker of adiposity and cardio-metabolic disease risk.

Due to the large number of OTUs in the top-ranked association results belonging to the same genera and families, we also explored the peak results with respect to collapsed taxonomies, whereby we combined sequences from OTUs with the same taxonomy. We first tested the association between the 8 genera, to which the 97 adiposity-significant OTUs were assigned, and adiposity. Altogether, 11 associations passed Bonferroni correction (*P* = 0.001), and 27 genus-adiposity associations were nominally significant. These comprised 6 of the genera and included *Blautia*, *Oscillospira*, *Lachnospira*, *Ruminococcus* (within both *Lachnospiraceae* and *Ruminococcaceae*) and *Clostridium*. The two most significant associations were obtained between *Oscillospira* and VFM (*P* = 3.29 × 10^−07^, Additional file 1: Table S3) and *Blautia* and VFM (*P* = 3.65 × 10^−06^). We considered a similar approach at the family level where the most significant bacterial-adiposity association was a negative relationship between *Christensenellaceae* and VFM (*P* = 1.48 × 10^−10^), supporting prior findings from Goodrich et al. [31].

We then explored fecal microbiome associations with obesity within a subsample of 247 MZ twin pairs to identify potential environmental or stochastic effects. MZ twins are matched for sex and age, and have nearly identical genomes and very similar early life environments. Therefore, microbiome differences observed within MZ twin pairs are likely to be a result of environmental and stochastic influences or effects that are secondary to the phenotype. We estimated differences in fecal OTU relative abundances within 247 MZ twin pairs and compared these to differences in adiposity, for each of the six adiposity phenotypes using a Pearson correlation. Although no results surpassed Bonferroni significance for multiple testing across the six phenotypes, potentially in part due to the smaller sample size, many of the observed effects were consistent with those observed across 1313 individuals (Additional file 1: Table S2). The peak association in MZ twin pairs was observed between an unknown *Clostridiales* OTU (Greengenes OTU 331113) and AGR (Pearson coefficient = −0.24, *P* = 9.68 × 10^−05^).

We performed two additional follow-up analyses of the association between fecal microbiome OTUs and adiposity phenotypes. Because of the high correlation across multiple obesity measures, we explored the association between fecal microbiome OTUs and adiposity measures independent of BMI. Following adjustment for BMI, 133 of the 149 significant associations remained nominally significant with the same direction of effect (Additional file 1: Table S2). Because of the strong association that we observed between alpha diversity and adiposity, we also wanted to assess if the strongest adiposity-OTU associations were with OTUs that were markers of diversity, or whether these taxa associated with adiposity were independent of species richness. To this end we repeated the adiposity-OTU analyses at the 149 significant OTU-phenotype associations now including alpha diversity as a covariate in the linear model as previously described [34]. All of the reported significant associations remained nominally significant after adjustment for alpha diversity.

### Replication of microbial-obesity associations

We pursued replication of the 97 significant OTUs associated with visceral fat in 4286 independent samples from three additional population-based replication samples, including samples from the American Gut, Flemish Gut Flora Project (FGFP) and extended TwinsUK cohorts. Due to the lack of cohorts with both visceral fat measurements and gut microbiome data available, BMI was used as a surrogate phenotype for visceral fat in these analyses. In each replication cohort, individuals selected were of European descent, over the age of 20, and had a BMI ranging between 18.5 and 30 units, resulting in 2338 individuals from the American Gut cohort (USA), 917 individuals from the FGFP cohort (Belgium) and 1031 individuals from the extended TwinsUK cohort (UK) who were not included in the discovery TwinsUK sample. To account for the difference between the 16S rRNA gene sequences between the TwinsUK discovery sample and the replication datasets, OTUs in each replication dataset were picked using closed reference in the software Quantitative Insights Into Microbial Ecology (QIIME) [43] at 97 % using UCLUST [44] against the representative sequences for the 97 Bonferroni-significant OTUs associated with adiposity measures, to maximize similarity in datasets during replication. Further processing and downstream analyses took into account technical and lifestyle cohort-specific covariates to match as closely as possible the discovery sample and account for differences across cohorts (see Methods).

The 97 OTU-BMI associations were tested within each replication sample (Additional file 5), and the results were combined in a meta-analysis across the three replication samples (Additional file 1: Table S4). At a stringent Bonferroni significance threshold (*P* = 0.05/97), 8 OTUs robustly replicated with the same direction of effect across the three replication cohorts (Fig. 3b, Additional file 1: Table S4), excluding results with evidence for heterogeneity. Furthermore, 13 OTUs showed evidence for association with BMI that was stronger in the meta-analysis across all four population-based cohorts, compared to the discovery TwinsUK sample alone (Additional file 1: Table S4). At a more relaxed significance threshold (nominal significance and same direction of effect in at least 1 cohort), 49 OTUs showed the same direction of effect and nominally significant evidence for association with BMI in at least one of the replication samples (Additional file 1: Table S4). The 8 OTUs with robust evidence for replication included members of *Lachnospiraceae* and *Ruminococcaceae*, while the 49 OTUs included not just members of the *Lachnospiraceae* and *Ruminococcaceae* families, but also of *Christensenellaceae*, *Clostridiales*, *Bacteroidaceae* and *Rikenellaceae*.

### Microbial functional alterations in obesity

Given the strong associations observed between fecal microbiome variation and obesity phenotypes, we next wished to assess potential functional differences that may be the result of a fecal microbiome dysbiosis in obesity. To this end, we aimed to infer KEGG functions of the fecal microbes by using the software Phylogenetic Investigation of Communities by Reconstruction of Unobserved States (PICRUSt) to predict metagenomes for each sample based on the closed-reference 16S rRNA gene sequences. The PICRUSt analyses resulted in altogether 233 Kyoto Encyclopedia of Genes and Genomes (KEGG) pathways and 6909 KEGG orthologies (KOs) within our sample. We next aimed to determine the relationship between the inferred KEGG functions and adiposity. We adjusted the KEGG pathway scores obtained for each individual in our sample for technical covariates (see Methods) and performed association analyses of these functions with the adiposity measures using a linear mixed effects model as previously described. We also assessed KO differential abundance in high and low visceral fat individuals using Statistical Analysis of Metagenomic Profiles (STAMP) [45].

**Fig. 4:**
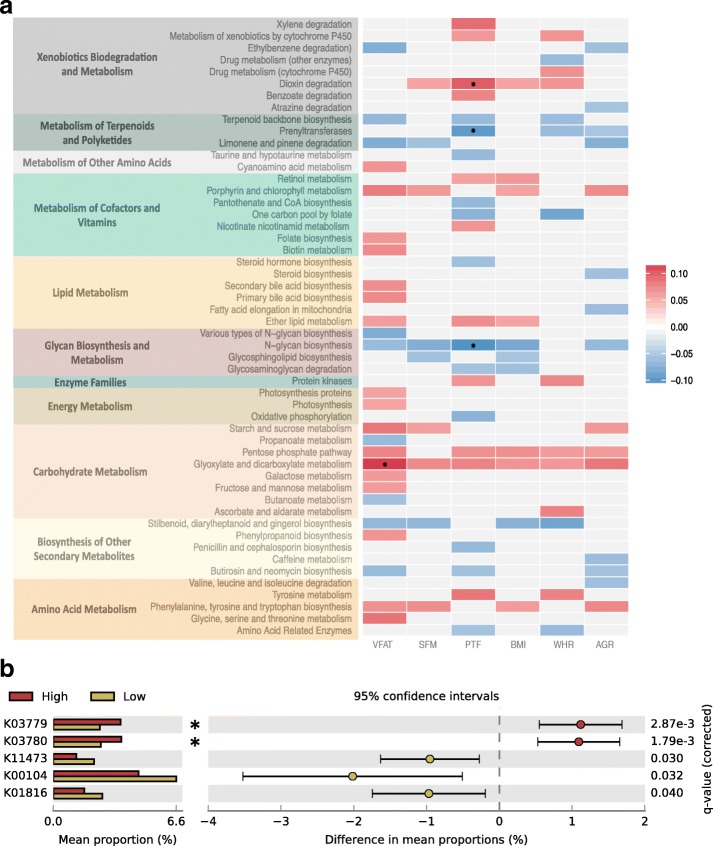
Microbial functional analysis in obesity. **a** Microbial PICRUSt-predicted KEGG functions relevant to metabolism in the twin dataset, and their association with the six adiposity measures. The *heatmap* denotes the direction of association between each microbial PICRUSt-predicted KEGG function and adiposity measures, where *blue* indicates a negative association, while *red* indicates a positive association. Bonferroni-significant associations are highlighted (*). **b** Five KO genes that are differentially abundant between high and low visceral fat individuals in glyoxylate and dicarboxylate metabolism, as tested by a two-sided Welch’s *t* test. FDR-adjusted *P* values are reported at the right of the image, and *stars* indicate Bonferroni-significant associations. Figure was produced using STAMP [45]

Of the 233 KEGG pathway associations with six adiposity measures, 13 associations surpassed Bonferroni correction (*P* = 3.6 × 10^−5^), and 218 associations were significant after false discovery rate (FDR) correction (FDR 5 %). Four of the 13 Bonferroni-significant associations were with KEGG functions related to metabolism, as well as 98 of the FDR 5 % significant results (Fig. 4a). Functions within carbohydrate metabolism were positively associated with adiposity, in particular, glyoxylate and dicarboxylate metabolism, which had a Bonferroni-significant association with VFM (*P* = 1.19 × 10^−06^) and FDR 5 % significant associations with the remaining adiposity measures. Five groups within the glyoxylate and dicarboxylate metabolism pathway were significantly differentially abundant in high visceral fat and low visceral fat individuals (Fig. 4b). Two of these five groups remained significant following Bonferroni correction, and these were K03779 (ttdA, *Q* = 2.87 × 10^−3^) and K03780 (ttdB, *Q* = 1.79 × 10^−3^); both increased in subjects with high visceral fat. The remaining 3 pathways that surpassed Bonferroni correction were obtained between pTF and dioxin degradation, prenyltransferases and N-glycan biosynthesis.

### Host genetic influences on microbiome-obesity associations

Twin-based heritability estimates supported a strong genetic component for visceral fat, and our heritability analyses of the fecal microbiome in this sample showed wide variability in heritability between fecal microbial taxa (0–0.42), with members of Firmicutes and Actinobacteria being most heritable. The average heritability of the overall fecal microbiome sample was 0.07, while the average shared environment component was 0.046 and the average unique environment was 0.93. The peak 97 Bonferroni-significant OTUs associated with abdominal adiposity in our sample had a median heritability of 0.16 and an average OTU heritability of 0.16. The average heritability of the 97 adiposity-associated OTUs was significantly greater than the overall average heritability over all OTUs at 0.07 (Wilcoxon rank test, *P* = < 2.2 × 10^−16^). In addition the average unique environmental component of the 97 is significantly lower than the overall average for all OTUs (0.79 versus 0.93, Wilcoxon rank test, *P* < 2.2 × 10^−16^). These estimates suggest host genetics impacts both the fecal microbiome and adiposity.

**Fig. 5:**
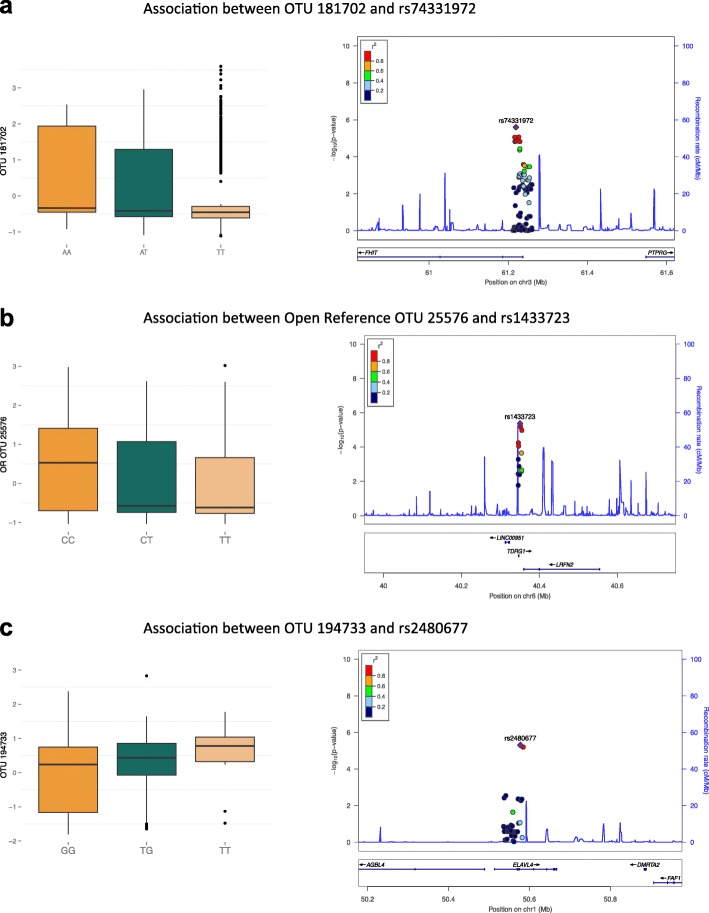
Peak genetic associations between obesity human genetic variants and adiposity-associated OTUs in the twin fecal microbiome. **a** Association between OTU 181702 and *FHIT* SNP rs74331972. The *boxplot* indicates change in OTU 181702 abundance with genotype at SNP rs74331972. The *LocusZoom plot* denotes the strength of association of OTU 181702 with SNP rs74331972, as well as the SNPs in the surrounding region. **b** Association between open reference OTU 25576 and *TDRG1* SNP rs1433723. The *boxplot* indicates change in open reference OTU 25576 abundance with genotype at SNP rs1433723. The *LocusZoom plot* denotes the strength of association of open reference OTU 25576 with SNP rs1433723, as well as the SNPs in the surrounding region. **c** Association between OTU 194733 and *ELAVL4* SNP rs2480677. The *boxplot* indicates change in OTU 194733 abundance with genotype at SNP rs2480677. The *LocusZoom plot* denotes the strength of association of OTU 194733 with SNP rs2480677, as well as the SNPs in the surrounding region. SNPs in all LocusZoom plots are coloured according to their strength of linkage disequilibrium with the peak SNP plotted

To explore the hypothesis that host genetics may influence the observed microbial-adiposity associations, we performed candidate gene analysis comparing host genetic variants at human obesity candidate loci with the adiposity-associated fecal microbiome profiles. We selected single nucleotide polymorphisms (SNPs) within human loci previously associated with obesity as reported by Locke et al. [8], using common genetic variants within 97 50-kb regions, centred around the peak BMI-associated GWAS SNP in each region. At a Bonferroni-corrected *P*-value threshold (*P* = 5.31 × 10^−06^) taking into account the total number of genomic regions and adiposity OTUs considered, OTU associations with genetic variants in three genomic regions surpassed multiple testing. The strongest association between host genotype and adiposity-associated OTUs was observed between an OTU within the *Clostridiales* order (Greengenes OTU 181702) and a host genetic variant within an intron of the *FHIT* gene (rs74331972 with OTU 181702, *P* = 2.49 × 10^−06^, Fig. 5a). *FHIT* encodes the fragile histidine triad protein and is a tumour suppressor gene that has been linked to cancers of the digestive tract. Although the most significant *FHIT* association was obtained with OTU 181702 (*h*^2^ = 0.13), which we identified as significantly associated with SFM and VFM (*P* = 1.18 × 10^−06^ and 1.27 × 10^−06^ respectively), the same genetic variant was also associated with another VFM- and SFM-associated OTU (rs74331972 with OTU 287790, *P* = 5.38 × 10^−05^). The second ranked significant genetic association was obtained between variants near gene *TDRG1* (peak SNP rs1433723, *P* = 4.32 × 10^−06^) with an open reference unknown *Clostridiales* OTU (*h*^2^ = 0.14), which we had identified as significantly associated with VFM (*P* = 4.97 × 10^−07^, Fig. 5b). The final significant genetic association was observed at a variant in an intron of the gene *ELAVL4* (rs2480677, *P* = 4.95 × 10^−06^, Fig. 5c) with an unknown *Blautia* OTU, 194733 (*h*^2^ = 0.02), which we had identified as significantly associated with VFM (*P* = 1.27 × 10^−07^), SFM (*P* = 2.26 × 10^−06^) and AGR (*P* = 3.11 × 10^−07^) in the peak 149 adiposity-OTU results. When we considered the genetic-OTU association results at a less conservative significance threshold (*P* = 5 × 10^−4^), there were in total 412 suggestive OTU-genetic associations located within or near 48 unique genes, including obesity genes such as *FTO*, *RPTOR* and *TMEM18*.

### Functional characterization of host genetic variants associated with adiposity-OTU markers in the gut

We next focused on the three significant host genetic associations in *FHIT*, *TDRG1* and *ELAVL4* with adiposity-associated OTUs. The transcriptomic profiles for these genes from the Genotype-Tissue Expression (GTEx) resource [46] indicated that all three genes are expressed in tissues that form part of the gastrointestinal tract. *FHIT* is expressed across a wide range of tissues, including stomach, colon (transverse and sigmoid), small intestine (terminal ileum), oesophagus (muscularis and mucosa) and oesophagus-gastro-oesophageal junction. *TDRG1* is expressed at highest levels in testis, in multiple brain tissues, as well as oesophagus (mucosa), while *ELAVL4* is expressed at highest levels in multiple brain tissues, testis, pituitary gland, colon (sigmoid and transverse), pancreas, small intestine (terminal ileum), oesophagus (muscularis) and oesophagus-gastro-oesophageal junction.

We next explored the functional impact of the SNPs in these three genes that we identified as most significantly associated with adiposity-related fecal OTUs. The GTEx expression quantitative trait locus (eQTL) analysis results indicate that rs1433723 in *TDRG1* is an eQTL for *TDRG1* expression specifically in oesophagus mucosa in the GTEx dataset. We then tested the associations of this variant on *TDRG1* gene expression and DNA methylation levels in adipose biopsies available for 542 individuals from the TwinsUK cohort [47, 48]. We found that rs1433723 also is significantly associated with DNA methylation levels in *TDRG1* (peak association: rs1433723 with *TDRG1* cg10553343, *P* = 1.53 × 10^–17^; Additional file 1: Table S5), but not with gene expression profiles in these samples in adipose tissue [47]. The remaining two associated SNPs with adiposity OTUs (rs74331972 in *FHIT* and rs2480677 in *ELAVL4*) were not associated with the corresponding gene’s expression levels in GTEx across multiple tissues. However, both variants showed modest effects on DNA methylation levels in adipose tissue (rs74331972 on *FHIT* cg15570148, *P* = 3.4 × 10^−4^; and rs2480677 on ELAVL4 cg00322486, *P* = 1.4 × 10^−3^), but not on gene expression profiles in adipose tissue in our dataset.

## Discussion

Here, in the largest microbiota-obesity study to date using detailed adiposity and visceral fat measures, we have shown that fecal microbial diversity and specific members of the human fecal microbiota are strongly associated with obesity-related phenotypes, specifically abdominal adiposity. The majority of microbial associations were obtained with visceral fat, a key metabolic disease risk factor, which we also show is strongly heritable in our extended sample of more than 3000 twins. In addition to obtaining novel heritability estimates for visceral fat, we show that android/gynoid ratio is highly heritable in this same cohort, and confirm high heritability estimates for the remaining adiposity phenotypes. Using BMI as a measure of obesity, we robustly replicate eight obesity-associated fecal microbes in three independent samples from the American Gut, FGFP and extended TwinsUK dataset. We also demonstrate that host genes have an effect on components of the fecal microbiota, including the 97 adiposity-associated OTUs, although the mechanism remains unclear. Given the impact of host genetics on both obesity and fecal microbes, and our findings of strong association between adiposity and fecal microbiome variation, our results therefore support the hypothesis that heritable microbes play a role in determining components of obesity relevant to cardio-metabolic disease and may be one potential source contributing to missing heritability in obesity.

### Heritability and importance of visceral fat

Visceral fat, the type of adipose tissue with the most important implications for metabolic health [2], was highly heritable (0.70) and showed the most significant associations with the fecal microbiota. Previous studies, for example, from the Framingham [39], Quebec [41] and Heritage [40] family-based cohorts, used CT scans to estimate visceral adiposity and found heritability estimates of visceral fat to be between 0.36 and 0.55, which is lower but comparable to our estimates in twins. Given the strong correlation between multiple adiposity phenotypes, we sought to assess if the high heritability observed in visceral fat is due to high heritability of BMI. Heritability of visceral fat remained high after adjustment for BMI (0.64, see Additional file 2), suggesting that the high heritability we report here for VFM is independent of its association with BMI.

Several microbiome studies have linked increased overall abdominal adiposity, for example, using body fat distribution measures such as waist/hip ratio, to fecal microbiome profiles [15, 49]. However, although specific probiotic intake has been reported to lower visceral fat [50], to date there has not yet been a systematic comparison of visceral fat and variation in the fecal microbiome across human subjects. Our results are therefore the first, to our knowledge, to link visceral fat with changes in the fecal microbiota variation in humans. The findings suggest that visceral fat mass is more important for differences in the obese microbiome, rather than overall body mass. This is also demonstrated in the recent Clarke et al. study [51], where elite rugby union players, who due to their greater muscle mass were defined as overweight and obese (BMI = 29 ± 3), had a more diverse microbiota than those of both low BMI and high BMI controls. We also report a novel android/gynoid ratio heritability estimate of 0.65, higher than previous family-based estimates of 0.43 [52]. Android/gynoid ratio, like visceral fat, is also a risk factor for cardio-metabolic disease, and accordingly our results show consistent direction of association effects for OTUs associated with both phenotypes.

### Visceral fat has widespread associations with the human fecal microbiome

Due to its importance in cardio-metabolic disease risk, direct measures of visceral fat are much more informative than BMI in assessing the metabolic consequences of obesity. Most microbiome obesity studies to date have used BMI as a biomarker, with some mouse studies using epididymal fat. We therefore present a novel dual approach, using visceral fat to find novel metabolic associations in a sample of twins, and BMI to confirm associations across independent samples. The power of this design was apparent in the number of peak associations observed with visceral fat. All of the reported adiposity OTUs reported in this study were significantly associated with visceral fat, while only 7 of these OTUs were significantly associated with BMI, suggesting that microbial studies that only use BMI as a measurement of obesity may be limited.

Altogether, we identified 97 OTUs that were strongly significantly associated with the adiposity phenotypes, and all of these were significantly associated with visceral fat. Although the 97 OTUs were not the most abundant OTUs within the gut, OTUs within the Firmicutes phylum showed the most significant adiposity associations, perhaps in part reflecting the dominant frequency of Firmicutes in the human gut. However, the direction of association differed for different genera within the Firmicutes. For example, *Oscillospira* OTUs often showed protective associations with VFM, while *Blautia* OTUs commonly showed adverse associations that could be used as potential markers of cardiovascular risk. Our findings also confirm the importance of fecal microbiome diversity in obesity, as we are able to replicate strong association between adiposity phenotypes and alpha diversity. Furthermore, the majority of the observed 97 VFM associations are independent of alpha diversity and BMI, suggesting that the OTUs significant in this analysis are not just markers of diversity, but form real associations with adiposity and cardio-metabolic disease risk.

To explore the factors underlying the variation in the 97 adiposity-associated OTUs, we used twin modelling and determined that these 97 OTUs showed significantly greater average heritability (0.16) compared to all OTUs in the larger dataset. The average shared environment component was also higher, but the average unique environment component was lower than the overall dataset average. We therefore infer that host genetics in particular, as well as early shared environmental factors, play an important role in the variation of obesity-associated microbes. However, although the estimate (including measurement error) is approximately 0.80, the average unique environment component in the 97 OTUs was lower than the overall dataset average. This is consistent with findings from previous work [31] that environmental factors are key drivers of the microbiome, and they strongly contribute to variation at the obesity-microbiome associations.

As our results consisted of OTU associations within the same families and genera of bacteria, we also explored associations of adiposity at the collapsed taxonomic level (family and genus) for the top OTU associations. *Lachnospiraceae* and *Ruminococcaceae* continue to display opposing directions of effect at the family level. We also see that the heritable family *Christensenellaceae* is strongly associated with visceral fat, as previously reported for BMI in Goodrich et al. [31]. *Christensenellaceae* was previously found to be protective of obesity both in mice and in human twins in our previous work [31]. We extend the findings here, showing strong protective associations between *Christensenellaceae* and visceral fat, in particular suggesting that individuals with *Christensenellaceae* have less cardiovascular risk than those without. Further work to elucidate the mechanism of protection is required.

### Partial replication of adiposity associations using BMI in independent cohorts

We pursued replication of the fecal microbial adiposity associations in a large sample of 4286 Caucasian individuals from three population-based cohorts. Due to the lack of cohorts with both visceral fat measurements and gut microbiome data available, BMI was used as a surrogate measure for visceral fat. Therefore, the findings in this section do not capture strict replication, but rather validation of the visceral fat fecal microbial associations. At a stringent Bonferroni threshold 8 OTUs replicated across all cohorts, and at a more relaxed threshold 49 OTUs replicated at nominal significance in at least one cohort with the same direction of effect as in the discovery sample. The replication results included OTUs classified within *Lachnospiraceae* and *Ruminococcaceae*. A higher relative abundance of Firmicutes has previously been associated with obesity in some but not all studies, and several of these studies show an increase in Firmicutes in obese subjects [12, 53, 54]. For example, *Ruminococcus gnavus* has been shown to be significantly enriched in low microbial gene count individuals who were prone to obesity in one study [15]. However, the phylum Firmicutes contains more than 270 genera with many different and diverse functions. Because changes may occur at finer-grained taxonomic resolution, and because the baseline abundance of different genera differs among human populations, opposing selection processes at different phylogenetic levels may in part explain differing results in studies determining microbiota differences in obesity [55, 56], including recent meta-analyses across multiple obesity microbiota datasets [16, 57]. This difference in directional effects in genera of the same family has been noted in animal models. Mice consuming a Western diet have previously been reported to have increased levels of *Eubacterium dolichum* [58], while another study has shown that mice consuming a high-fat diet have decreased levels of *Allobaculum* OTUs [59]. Both of these microbes are members of the *Erysipelotrichaceae* family, and yet they show opposite directions of effect in our data.

We observe a small number of robust associations across all cohorts, but more than half of the findings validated in at least one of the replication cohorts. The reasons for the partial replication of our results are unclear and are likely in part to capture differences in protocols, genetics, geography, lifestyle and diet, which are difficult to account for in full during the analysis. The American Gut dataset used the protocols developed for the Earth Microbiome Project [60], which are largely the same as those used in Goodrich et al. [31], except that the American Gut dataset is filtered to remove *Gammaproteobacteria* sequences increased following transit. FGFP protocols are most similar to those of TUK-D and TUK-R, however, with similar sample collection methods and sequencing protocols. Analysis of the American Gut data adjusted for more covariates than TUK-R, TUK-D and FGFP, and the cohort itself included a different age range (average 50, 21–94) and gender ratio (53 % female). The age range and gender ratio of the FGFP cohort were similar to those of AG (average age = 51 (21–85), 55 % female), while TUK-R was expectedly more similar to TUK-D (average age = 57.3 (20–89), 80 % female). Additionally, the American Gut samples were primarily from the USA (84 %), unlike the discovery and replication TwinsUK samples from the UK and the Belgian FGFP samples. The majority of the 97 OTUs show the same direction of association in the TwinsUK discovery, TwinsUK replication and FGFP samples, but this is not the case in the American Gut sample results (Additional file 5). This observation may partly reflect potential differences in OTU associations in different populations and geographical locations, specifically between European and American samples. Previous studies have shown some differences at the OTU level between countries [42, 61]. The sample collection methods also differed between the European studies with greater quantities collected in TUK-D, TUK-R and FGFP compared to swabs in American Gut, although neither used fixatives. These factors highlight the on-going difficulty in replicating OTU level results between studies and cohorts. Additional standardisation of both technical and analytical methods will help distinguish features common across populations, those sensitive to technical influence and those unique to a particular region or group.

### Microbial metabolism is altered in obese individuals

Our findings of strong association between particular microbes along with their directional effect in obesity can provide insights into functional impacts. It has been suggested that the dysbiosis of microbial species in obesity is not as important as the resulting functional dysbiosis [62]. Using the PICRUSt method to predict metagenomes and functions, we find an enrichment of metabolism-related KEGG functions associated with adiposity, in particular, carbohydrate metabolism and N-glycan biosynthesis. We see a strong positive association between visceral fat and glyoxylate and dicarboxylate metabolism, particularly with the genes *ttdA* and *ttdB*. The glyoxylate cycle, a pathway that until recently was thought to be absent in most animals [63], is able to metabolise fatty acids into glucose, thus contributing to insulin resistance in the event of fatty acid abundance [64]. More recently glyoxylate has been highlighted as a biomarker of type 2 diabetes, even as much as 3 years prior to diagnosis of diabetes [65]. Here we show a potential mechanistic impact of the microbial dysbiosis observed in individuals with increased visceral fat. We cannot assess whether the heritable microbes are driving the differences observed in these pathways; however, it would be interesting to determine the heritability of the inferred microbial functions, particularly in future studies with metagenomic datasets.

### Host genetics influences the human fecal microbiome and its link to central adiposity

Our new OTU heritability estimates in this larger twin sample remain largely similar to those in the original Goodrich et al. [31] twin-based heritability analysis results, although peak heritable OTUs are slightly different. Comparison of the heritability results at the genus level show that consistent estimates were obtained across the two datasets (*r* = 0.67, Additional file 3: Figure A), and these were also consistent with a recent heritability analysis of the extended TwinsUK dataset [33] (*r* = 0.77, Additional file 3: Figure B). The most heritable taxon in our dataset is an OTU classified as *Clostridium perfringens* (*h*^2^ = 0.42). The heritability estimate for the most heritable OTU reported in the Goodrich et al. [31] study, OTU 176318 assigned to *Christensenellaceae* (original heritability estimated at 0.36), remained similar in the extended dataset, with a new heritability of 0.31. The most heritable OTUs in our dataset were relatively frequent, being present in at least 25 % of individuals. A potential mechanism that underlies fecal microbial heritability is the ability of the host genome to impact the gut environment, for example, by altering acidity of the gut or by host control of microRNAs that can enter bacterial cells and affect bacterial growth [66]. Therefore, the likely mechanism of microbial heritability is host genetic manipulation of microbes [67].

The OTUs that we identified as significantly associated with adiposity phenotypes displayed significantly greater estimates of heritability compared to all OTUs profiled in the gut, which therefore prompted us to test if specific human genetic variants may explain the observed microbial heritability at these 97 adiposity-associated OTUs. To explore how human genes could influence these microbes in the context of obesity, we pursued host genetic association analyses of the adiposity-associated OTUs using a candidate gene analysis approach at host obesity GWAS loci. The most significant genetic association was obtained with a variant in the gene *FHIT*, which encodes the fragile histidine triad protein. Significantly reduced *FHIT* gene expression levels have been reported in cardiac tissue from obese compared to lean subjects [68]. Although this genetic variant is not an eQTL itself, it does show moderate evidence for genetic impacts on DNA methylation levels in adipose tissue, specifically at a CpG site in a CpG island shore in the promoter of the *FHIT* gene. Therefore, it may have an indirect impact on *FHIT* adipose tissue gene expression in obesity through epigenetic regulation of *FHIT* expression. *FHIT* also appears to act as a tumour suppressor in several types of cancer [69], and its abnormal function has been linked to cancers of the digestive tract [70]. The *FHIT*-associated OTU is strongly associated with abdominal adiposity in our data, including SFM and VFM, suggesting that it plays a role in cardio-metabolic disease risk in the host.

Human genetic variants in or near two other genes, *ELAVL4* and *TDRG1,* were also significantly associated with adiposity OTUs. Although these genes currently lack a clear biological link to obesity, they are expressed in tissues that are part of the GI tract. Furthermore, rs1433723 in *TDRG1* is an interesting candidate for follow-up, as it exhibits strong genetic influences on *TDRG1* gene expression in parts of the GI tract (oesophagus mucosa). This genetic variant also impacts *TDRG1* DNA methylation levels in adipose tissue, specifically at a CpG site in an intra-genic CpG island in the gene body, which could mark an alternative transcription start site. GTEx summary expression profiles of this gene correspondingly show exon bias in expression levels across different tissues, consistent with alternative transcript usage.

The four significantly associated host genetic variants in *FHIT*, *ELAVL4* and *TDRG1* were not the same as the lead SNPs for each candidate obesity locus reported in Locke et al. [8], and are in low to moderate linkage disequlibrium (LD) with the corresponding lead SNPs (*r*^2^ = 0.014–0.25). This could be attributed to several reasons, including sample size and imputation differences. Compared to our sample of 1313 twins, the sample size in Locke et al. [8] is more than 300,000 individuals, resulting in excellent power to detect phenotypic associations with particular lead SNPs. There were also differences between the imputation strategies used by the two studies; whereas in Locke et al. [8] SNPs were imputed to the HapMap 2 reference panel, our dataset was imputed to the 1000 Genomes reference panel. These and other factors may impact the association of the lead SNP from Locke et al. [8] with OTUs in this dataset.

Our heritability and host genetic association results show that human genetic factors have a role both in determining obesity and in influencing the host fecal microbial composition. The strong associations that we detect between fecal microbes and obesity phenotypes suggest that host genetics may influence these microbial-obesity associations. Using a candidate gene approach, we identified host genetic variants in three obesity human loci which show associations with adiposity-associated fecal microbes in our dataset. These variants are promising candidates for further follow-up studies to assess their potential role in obesity-microbial interactions. Although human obesity is highly heritable, human genetic variants detected to date explain only a small proportion of the heritability in obesity. Given our observation that the average microbial heritability is greater at the most associated adiposity OTUs, our results are therefore consistent with the hypothesis that a proportion of the heritability in obesity may be explained by heritable fecal microbes.

### Limitations and considerations

Due to the cross-sectional and observational nature of the study, we are unable to determine causal relationships between the fecal microbiota, host genetics and visceral adiposity. Furthermore, to determine more robust genetic associations, we require a much larger sample size than is represented here. Lack of metagenomic data limits our functional interpretation of the microbial dysbiosis observed in obesity, although predictions from PICRUSt do provide some interesting insights for further investigation. Some research has shown that in individuals who are dieting, there is an increase in Bacteroidetes and, conversely, individuals who are over-eating show an abundance of Firmicutes [53]. It is therefore important to know whether the individuals in the study were calorie restricted. This is one limitation of the present study, and future work should focus specifically on how the interaction of diet affects visceral fat associations with the fecal microbiome. Other considerations that should be taken into account in the future are stool consistency [71] and antibiotic and other drug use [72, 73].

Due to the novelty of visceral fat measurement and lack of adiposity measurements other than BMI in other microbiome datasets, we lacked true replication in independent cohorts, which would likely have improved our results, as would have having a better balance of genders in discovery and replication samples.

## Conclusions

We have found strong associations between fecal microbial profiles with total and visceral fat, in the largest fecal microbiota-obesity study to date using multiple measures of human adiposity, some of which are robustly replicated. We identify novel and confirm previously established fecal microbe associations with overall obesity. Additionally, our study can help distinguish protective and risk microbes in human cardiovascular and metabolic disease risk by using visceral fat as risk phenotype. We obtain novel high heritability estimates for visceral fat and android/gynoid ratio in a large twin sample, and confirm previously reported heritability estimates for other adiposity measures. Furthermore, we identify promising host genetic variants that may influence the interaction between the human fecal microbiome and obesity and its metabolic consequences. Our findings support the hypothesis that heritable microbes play a major role in the components of adiposity that are most relevant to cardio-metabolic disease risk.

## Methods

### Subjects and sample collection

All subjects included in the study were healthy volunteers from the TwinsUK Adult Twin Registry [48, 74]. Adiposity phenotype data were collected on the extended sample of 3666 twins (Table 1). These included 1044 monozygotic (MZ) and 789 dizygotic (DZ) predominantly female twin pairs. The sample participants were predominantly of European descent and the average age was 63 (Table 1). Fecal microbiome data were obtained for a subset of 1313 individuals from the sample 3666 twins. The microbiome dataset included 496 MZ, 594 DZ and 223 unrelated individuals, who were also predominantly female and European, and the average age was also 63 (Table 1 and Additional file 1: Table S7). Fecal samples from all individuals followed the same collection protocol as previously reported [31, 33, 34]. Briefly, fecal samples were refrigerated or kept on ice for 1–2 days prior to arriving at the laboratory at the Department for Twin Research, King’s College London, at which point they were immediately stored for up to 8 weeks at −80 °C before DNA extraction. Frozen samples were shipped to Cornell University for DNA extraction, PCR amplification and sequencing. The study was approved by the local research ethics committee, and signed and written consent was obtained from all participants.

### Fecal microbiome profiles

This manuscript explores 16S gut microbial profiles from a total of 1313 twin stool samples. Of these, 601 16S fecal microbiome profiles were previously described by Goodrich et al. [33], 671 profiles were recently published in Jackson et al. [34] and additional 16S microbial profiles were generated in a further 41 twin stool samples [33], giving us altogether 1313 individuals for whom both 16S gut microbial data and adiposity measures were available. The sequence data for the discovery TwinsUK fecal microbial sequence dataset used in this study are available from the European Nucleotide Archive (ERP006339, ERP006342, ERP015317. Sample accessions can be found in Additional file 1: Table S8).

Briefly, all samples underwent the same laboratory protocol and data processing steps, following the quality control procedure outlined by Goodrich et al. [33]. DNA extracted from fecal samples underwent amplification of the V4 region of the 16S rRNA gene using the 515F and 806R primers, followed by 250-bp paired-end sequencing on the Illumina MiSeq platform. Processing of sequencing and OTU picking was carried out as previously described [34]. In brief, paired-end sequences were merged with at least a 200-bp overlap, and those longer than 275 bp were filtered from the dataset. The remaining sequences were analysed using QIIME 1.7.0 (Quantitative Insights Into Microbial Ecology) [43]. Sequences containing ambiguous or low quality reads (Phred score ≤25) and uncorrectable barcodes were removed from the dataset and open-reference OTU picking was performed against the Greengenes May 2013 database. OTUs not found in at least 25 % of individuals were then discarded, and their counts were converted to relative abundances within samples, followed by the addition of a pseudocount (10^−6^) to remove zero counts. Mixed effects models were fitted to these data to control for technical and batch effects. The regression included sequencing run, depth of sample sequencing and technician who extracted and loaded the DNA for sequencing, as well as sample collection method (by post or in person) as predictors with OTU abundances as the response. Residuals from these models were then used in subsequent downstream analyses. The adiposity OTU and OTU candidate gene downstream analyses also included further adjustment for covariates such as age, gender and five long-term (10-year) summary broad dietary profiles (see following subsection on phenotype data). The final dataset contained 2135 OTUs.

In estimating collapsed taxonomy quantifications, OTUs from the complete set (including those in <25 % of individuals) were collapsed into genera and families based on shared taxonomic assignment. Taxa found in fewer than 10 individuals were then discarded and the counts converted to relative abundances. We applied the OTU quality control regression framework to correct for technical covariates and batch effects as described above, and then used the resulting residuals in subsequent downstream analyses.

### Phenotype data

Obesity phenotype data were collected during each participant’s annual clinic visit. We explored six obesity-related phenotypes in this work for a complete dataset of 3666 phenotyped twins. The adiposity phenotypes included a number of measures from total body dual-energy X-ray absorptiometry (DXA) whole-body scanning. During the DXA procedure participants were asked to lie flat and straight while a full body scan took place, as previously reported [75], taking measures for percentage trunk fat and visceral fat mass (g) [32]. Visceral fat mass was calculated from one cross section of the whole body at L4–L5, the typical location of a CT slice. Other phenotypes collected were subcutaneous fat mass, percentage trunk fat, BMI, android/gynoid ratio and waist/hip ratio. Subjects were asked to remove their shoes and height (cm) was measured using a stadiometer. Weight (kg) was measured on digital scales. Waist circumference was measured using a tape, halfway between the lower border of the ribs and the iliac crest in a horizontal plane. Hip circumference was measured at the widest point over the buttocks.

Covariates for phenotypic analyses included age and gender in all 3666 individuals and dietary profiles in 1313 individuals. We did not have detailed dietary information at the time of fecal sample donation for this dataset; however, we had available previously collected and reported dietary profiles [76]. Teucher et al. collected food frequency questionnaires and performed a principal component analysis on these data. The proportion of variance explained by the first five principal components was 22 %, and they corresponded broadly to the following approximate diets: Fruit and Veg, Traditional English, High Alcohol, Dieting and Low Meat (see [76]). We used these five variables as long-term stable dietary profiles in the downstream microbiome association analyses.

### Statistical analysis

To assess the evidence for association between fecal microbiome composition and obesity-related and metabolic phenotypes, we performed two principal analyses. First, we compared microbiome OTUs and phenotypes by fitting a linear mixed effects regression (LMER) model using the R package lme4 [77] across all 1313 individuals. In this model the phenotype was the response variable, and the OTU was a fixed effect predictor. Additional factors included family and zygosity taken into account as random effects, and sex, age and dietary profiles (principal components) considered as fixed effects. Each phenotype was normalised to a standard normal distribution prior to analysis. To assess the significance of the associations, we compared the full regression model described above to a null model that excluded the OTU predictor using an analysis of variance (ANOVA) test in R. We report associations that passed nominal significance (*P* = 0.05), as well as those that passed a Bonferroni threshold for multiple testing (*P* = 3.90 × 10^−06^).

Heritability, the proportion of total variance in a trait attributed to genetics, was assessed using the ACE model. Under the assumption that the dominance effects are negligible, the ACE model can estimate the additive genetic (A), common environment (C) and unique environment (E) components of the trait variance. Narrow-sense heritabilities were estimated from the proportion of the total phenotypic variance explained by estimated additive genetic effect. To estimate parameters of the ACE model, a maximum likelihood method was applied under multivariate normality assumptions using OpenMx software [78], a structural equation modelling package in R. We also provide 95 % confidence intervals for both phenotype and OTU heritability estimates in this study.

Functional analysis was performed using PICRUSt v1.0.0. Counts of KEGG functions were obtained and then transformed into relative abundances. The values were then transformed using the Hellinger transformation prior to adjustment for the following technical covariates: sequencing run, technician who performed the DNA extraction and technician who loaded the plate. The functional residuals from this adjustment were then fit into a linear mixed effects regression as predictor variables, where adiposity phenotypes were the response. Further covariates in the regression included age, sex, zygosity and family structure as described above. Differential abundance analysis was performed on the residuals using STAMP [45], whereby a two-sided Welch’s *t* test was used to test the difference in KO counts between high and low visceral fat groups. A Benjamini-Hochberg FDR correction [79] was applied to the association *P* values from the linear mixed effects regression and the differential abundance analysis.

### Host genomic analyses

Host genotype data were available in 1059 individuals from the microbiome dataset (see Additional file 2). Briefly, genotyping in TwinsUK was performed with a combination of Illumina HumanHap300, HumanHap610Q, 1MDuo and 1.2MDuo 1 M chips, and genotypes were called as previously described [48]. Imputation was performed using the IMPUTE software package (v2) [80] using as reference panel the 1000 Genomes haplotypes (based on SHAPEIT2) Phase I integrated variant set release (v3, September 2013). See Additional file 2 for further details.

In the candidate gene analysis the list of human candidate obesity GWAS SNP associations was obtained from Locke et al. [8]. The reported SNPs in Locke et al. were taken as the lead SNP, and candidate gene regions were extended to include additional SNPs within a 25-kb region either side of the lead SNP. SNPs were included in downstream analysis if they had a minor allele frequency (MAF) of 5 % and an info score of more than 0.4 in our imputed sample. Overall, there were 8876 SNPs across 97 unique genomic regions that were included in downstream association analyses. Host genetic association analysis was performed using the software Genome-wide Efficient Mixed Model Association (GEMMA) [81] at the variants included in the 97 human obesity candidate loci. GEMMA implements a univariate linear mixed model to perform association tests, using a kinship matrix to take into account twin relatedness.

DNA methylation profiles were obtained in 542 female Caucasian twins using the Infinium HumanMethylation450 BeadChip assay (Illumina 450 k). The DNA methylation Illumina 450 k dataset was obtained from adipose tissue biopsies in the twins, as previously described [48] (ArrayExpress E-MTAB-1866). DNA methylation levels were first normalised using BMIQ [82], and adjusted for covariates including age, smoking, alcohol, zygosity, family, plate, bisulphite-sequencing (BS) conversion efficiency and BS conversion concentration (see Additional file 2). The resulting residuals were normalised, and methylation QTL analysis was performed using Matrix eQTL [83]. The analysis considered genetic association under the additive model between genetic variants at rs74331972, rs1433723, rs2480677 and 467,928 DNA methylation probes that passed quality control.

Gene expression quantitative trait locus (eQTL) results were available from a large-scale study of human gene expression in multiple tissue samples including subcutaneous fat, lymphoblastoid cell lines and whole skin, derived from 856 monozygotic (MZ) and dizygotic (DZ) female twins from the TwinsUK cohort, as part of the MuTHER project [47]. We interrogated the candidate SNPs for eQTL results in adipose tissue (subcutaneous fat) using the Genevar software [84]. The functional impact of the candidate SNPs was also explored using GTEx results across multiple tissues, using GTEx Analysis Release v6 (dbGaP Accession phs000424.v6.p1) [46].

### Replication of obesity-microbial associations in independent cohorts

#### American Gut cohort

Replication analyses were pursued in 2338 individuals from the American Gut project (see Additional file 2). American Gut (AG) participants were selected from sequence rounds 1–21 (EBI: ERP012803) as Caucasian over the age of 20 with a BMI between 18.5 and 30, and living in the USA, UK, Australia or Canada (Additional file 1: Table S7). Sequencing protocols of the AG samples have previously been described [60, 85, 86]. Additionally, the fecal sample had to have at least 1000 sequences when picked closed reference using SortMeRNA [87] against the August 2013 release of Greengenes [88]. AG samples were pre-processed to remove candidate overgrowth sequences, as described elsewhere [89]. OTUs were picked using closed reference in QIIME [43] at 97 % using UCLUST [44] against the 97 replication OTUs, and 96 OTUs were successfully identified. Regression was performed in Statsmodels [90] using a three-step model. OTUs were power transformed and offset by 1. The power transform was regressed to control for technical covariates (see Additional file 2). Residuals from this were regressed against lifestyle covariates, including age, last antibiotic use, IBD diagnosis, flossing frequency and country. Normalised BMI was regressed against the residual from the second regression, with sex as a covariate. In the AG BMI-OTU association results we observed that 26 associations had the same direction of effect while 18 were nominally significant and had the same direction effect as the TwinsUK discovery sample.

#### Flemish Gut Flora Project cohort

Replication analyses were pursued in 917 individuals from the Belgian Flemish Gut Flora Project (FGFP) (see Additional file 2), selected as Caucasians over the age of 20 with a BMI between 18.5 and 30 [17]. Prior to OTU picking, sequencing depth was downsized to 10,000 reads per sample. OTUs were picked in QIIME [43] at 97 % using UCLUST [44] by closed reference picking against the 97 replication OTUs, and 96 OTUs were successfully detected in FGFP. OTU abundances were power transformed and offset by 1. The power transform was regressed to control for age, gender, alcohol average consumption in the week prior to sampling and smoking (yes/no). Normalised BMI was then regressed against the residual from the first regression, with gender and dietary restrictions (None/Vegetarian, Vegan, Macrobiotic/Other) as covariates.

In the FGFP BMI-OTU association results we observed that 83 associations had the same direction of effect while 34 were nominally significant and had the same direction effect as the TwinsUK discovery sample.

### TwinsUK replication dataset

The extended TwinsUK fecal microbiome dataset was recently described [33] and included a set of 1031 individuals (not overlapping with the 1313 TwinsUK discovery sample here) for whom both fecal microbial profiles and BMI, but not visceral fat, were available. We therefore included these additional data as a third replication sample (TwinsUK (TUK-R)), where all individuals were of European descent over the age of 20 with a BMI between 18.5 and 30. Read data were closed-reference clustered using the representative sequences for the replication OTUs as a reference, using UCLUST [44] at 97 % in QIIME v1.9.0 [43]. Counts for each OTU in each individual were converted to relative abundances by dividing by the total number of reads in the sample. A count of 0.000001 was added to the relative abundances to account for zeros, and these were then log transformed. The transformed counts were then residualised to adjust for sequencing run, collection method, person who loaded the plate and person who performed the DNA extraction. Lifestyle covariates included in downstream analyses matched those described for the TwinsUK discovery sample. Altogether, 76 associations showed the same direction of effect and 7 associations were nominally significant in the TwinsUK replication sample.

### Meta-analysis across cohorts

Random-effects meta-analysis was first performed across the three replication samples (AG, FGFP and TUK-R). We considered OTU-BMI results to replicate if they showed the same direction of association with BMI in the meta-analysis as in the discovery TwinsUK sample, and if the significance level in the meta-analysis passed Bonferroni adjustment for multiple testing (*P* = 5.15 × 10^−4^). We also performed a meta-analysis across all four population samples to identify additional OTUs at which the evidence for association with BMI improved over the discovery TwinsUK *P* value, even if they did not reach the Bonferroni significance cut-off for replication. In each meta-analysis, we assessed evidence for heterogeneity using Cochran’s Q statistic and the *I*^2^ statistic [91], and only considered results with no strong evidence for heterogeneity (Cochran’s Q *P* > 0.05 and *I*^2^ < 0.75). The meta-analysis was performed in R 3.1.2 with the R package ‘metafor’ [92] using as input beta coefficients and standard errors from each independent cohort.

## Additional files

Additional file 1: Supplementary Tables. **Table S1.** OTUs showing at least moderate (A >0.2) heritability in the extended TwinsUK 16S dataset. Each variance component, additive genetics (A, or heritability), shared environment (C) and unique environment (E) shown along with upper and lower 95 % confidence intervals. Average relative abundance of the OTUs in the overall dataset is also shown. **Table S2.** 149 Bonferroni-significant OTU-adiposity associations. OTU-adiposity results are obtained from linear mixed effects regression models and include the original results (OTU-Adiposity) and results after adjustment for BMI (OTU-Adiposity BMI-adjusted). We also provide the OTU heritability and average OTU abundance in the cohort. The last two columns show the OTU results from the within MZ twin-pair difference analyses (MZ Diff Beta), and whether the MZ results were concordant with the linear mixed effects results. Reported phenotypes are visceral fat (*VFM*), subcutaneous fat (*SFM*), android/gynoid ratio (*AGR*), BMI, waist/hip ratio (*WHR*) and % trunk fat (*pTF*). **Table S3.** Genus-level associations with adiposity, where the collapsed taxonomy OTU genus was significantly associated with adiposity. Reported phenotypes are visceral fat (*VFM*), subcutaneous fat (*SFM*), android/gynoid ratio (*AGR*) and BMI. **Table S4.** Replication of TwinsUK (*TUK-D*) results in three independent cohorts, the American Gut (*AG*), the Flemish Gut Flora Project (*FGFP*) and the expanded TwinsUK dataset (*TUK-R*). The table lists the 97 OTUs forming 149 significant adiposity associations in TwinsUK and their association with BMI in both the replication and discovery samples. In addition, the table outlines the results of two meta-analyses that were performed across the studies, the first of just the independent cohorts, and the second including the discovery cohort. Finally, the last section of the table shows which OTUs were replicated in at least one of the replication cohorts. **Table S5.** Human genomic analyses at adiposity-related candidate host genes *FHIT*, *TDRG1* and *ELAVL4*. The table shows significant genetic associations between the adiposity-related candidate host genetic variants and adiposity-associated OTUs. The final three columns summarize the results of the association between the host genetic variants and DNA methylation at CpG sites targeted by the Illumina 450 k array. DNA methylation association was performed at the lead OTU-associated SNP in each locus; therefore, rs1433722 was not tested (NA). **Table S6.** List of the 97 BMI-associated loci reported in Locke et al. [8] that were used for analysis in this study. The last column denotes the BMI GWAS *P* values from Locke et al. [8]. **Table S7.** Demographics of the TwinsUK microbiome discovery sample and the American Gut replication sample. **Table S8.** ENA accession IDs for samples in this study currently available online and basic metadata. (XLSX 252 kb) Additional file 2: Supplementary methods including expanded methods for analyses and data collection performed within the discovery cohort. (DOCX 168 kb) Additional file 3: Genus-level heritability estimates between the TUK discovery dataset and Goodrich. A) Scatterplot showing the genus-level heritability between TUK-D and Goodrich et al. [31] (*r* ^2^ = 0.67). B) Scatterplot showing the genus-level heritability between TUK-D and Goodrich et al. [33] (*r* ^2^ = 0.76). (PDF 43 kb) Additional file 4: Comparison of alpha diversity and human adiposity phenotypes. Alpha diversity is measured using the Shannon metric. For each adiposity phenotype considered in this study we present the *r* ^2^ and strength of association, and a trend line is shown in *red*. (PDF 693 kb) Additional file 5: Concordance of OTU-BMI association results at the 97 OTUs between TwinsUK discovery sample and the three independent replication cohorts. Points marked in *red* show no consistent effect between the studies, while *blue* denotes consistent direction of association. *Blue points* that are *empty circles* are not significant, while *blue filled circles* indicate nominally significant results. A) Scatterplot between TwinsUK discovery sample (*TUK-D*) and American Gut (*AG*). B) Scatterplot between TUK-D and FGFP. C) Scatterplot between TUK-D and TwinsUK replication sample (*TUK-R*). (PDF 146 kb)
