# Supplementary material for: Heritable components of the human fecal microbiome are associated with visceral fat
Source: Genome Biol. 2016 Sep 26;17:189. doi: 10.1186/s13059-016-1052-7 (PMC5036307; doi:10.1186/s13059-016-1052-7)
Supplement: Additional file 2: — Supplementary methods including expanded methods for analyses and data collection performed within the discovery cohort. (DOCX 168 kb) [file 13059_2016_1052_MOESM2_ESM.docx]

Supplementary Materials

Sample processing and DNA extraction

TwinsUK (Discovery Cohort)

Faecal samples were collected as previously described[1, 2]. Twins produced faecal sample at home. The sample was then refrigerated and/or kept on ice before it arrived in the Department of Twin Research laboratory, at King’s College London - where it was kept frozen at -80°C. Frozen samples were shipped to Cornell University where DNA extraction, PCR amplification, and sequencing took place.

Genomic DNA was isolated from an aliquot of ∼100 mg from each sample using the PowerSoil® - htp DNA isolation kit (MoBio Laboratories Ltd, Carlsbad, CA). 16S rRNA genes were amplified by PCR from each of the samples using the 515F and 806R primers for the V4 hypervariable region as previously described[3]. PCR reactions, carried out in duplicate, consisted of 2.5 U Easy-A high-fidelity enzyme, 1 × buffer (Stratagene, La Jolla, CA), 10-100 ng DNA template, and 0.05 μM of each primer. Reaction conditions consisted of initial denaturation at 94°C for 3 min followed by 25 cycles of denaturation at 94°C for 45 s, annealing at 50°C for 60 s, extension at 72°C for 90 s, and a final extension at 72°C for 10 min. The replicate PCR reactions were combined and purified using a magnetic bead system (Mag-Bind® EZPure, Omega Bio-Tek, Norcross, GA). PCR amplicons were quantified using the QuantiT PicoGreen dsDNA Assay Kit (Invitrogen, Carlsbad, CA). Aliquots of amplicons (at equal masses) were combined for a final concentration of approximately 15 ng/μl. DNA was sequenced using the Illumina MiSeq 2x250 bp platform at Cornell Biotechnology Resource Center Genomics Facility.

Replication Cohorts

In the American Gut samples were sequenced using the Earth Microbiome Project (EMP) protocols as previously described [4], using 515F/806R primers[5] or modified 515F/806R primers[6]. Multiplexed samples were sequenced using an Illumina MiSeq (La Jolla, CA). Technical covariates included in the first step of the regression analyses for the replication sample covered variables adjusting for sequencing depth, run, machine, processing robot, and extraction kit.

In the FGFP the V4 region was sequenced using 515F/806R primers as previously described[7], modified to contain Illumina adapters and barcode sequences to allow for directional sequencing. Paired-end sequencing was performed on the Illumina MiSeq platform and resulting reads were adjusted for technical and lifestyle covariates. Covariates included age, gender, alcohol average consumption in the week prior to sampling, smoking(yes/no), gender, dietary restrictions (None/Vegetarian,Vegan,Macrobiotic/Other).

In the TwinsUK replication sample the sequencing protocol and the technical and lifestyle covariates included in the downstream analyses matched those described for the TwinsUK discovery sample.

Sample descriptions

Supplementary Table 7 outlines the descriptive statistics of the TwinsUK discovery sample, and the three replication samples, listing information known at the time of analysis. No drug or biochemical measures were available for the TwinsUK and American Gut samples at the time of analysis and as such this information is not provided. In addition, Bristol Stool Scores are unavailable for the TwinsUK and American Gut samples.

Heritability of Visceral Fat following BMI-adjustment

In order to assess if heritability of visceral fat is driven by the high heritability of BMI, we adjusted visceral fat measurements for BMI in 3,666 subjects and repeated the heritability analyses. We used a linear model to adjust for BMI and took the residuals from this model into the new heritability analyses. We then performed univariate twin modeling on the residuals in OpenMx to estimate A, C and E. The results for visceral fat adjusted for BMI were as follows:

A = 0.64

C = 0.06

E = 0.3

Dietary Profiles

Dietary information was available for a subset of individuals in the Twins UK cohort. Full and extensive methodology describing these data can be found in Teucher et al (2007)[8], and a summary is included below.

Subjects completed a food frequency questionnaire (FFQ) that had previously been used and validated[9]. Here subjects were asked to indicate how often and how large a serving they had per week of 131 food items. A nutrient database was used to determine nutrient intake from each questionnaire and subjects were excluded from further analysis if

1/ they had left answers for more than 10 food items blank,

2/ the ratio of the FFQ derived estimate of total energy intake to the subject’s estimated basal metabolic rate (based on the Harris-Benedict equation[10]) was outside two standard deviations from the mean of that ratio (< 0.52 or > 2.58), and

3/ the subject’s twin had not completed an eligible FFQ.

For each food group, the frequency of intake (servings/week) was adjusted for total energy intake, using the residual method[11]. The energy-adjusted intakes were then standardized to z scores, and principle components analysis was performed on these scores. The first 5 PCs explained 22% of the variance in the dietary data and this was performed at two separate time points, 10 years apart. An average of the two PC datasets was taken and due to their rough adherence to actual dietary patterns, they were used for analysis in this study. The first 5 PCs can be described as below:

**Fruit and vegetable:**

Frequent intakes of fruit, allium and cruciferous vegetables; low intakes of fried potatoes.

**High alcohol:**

Frequent intakes of beer, wine and allium vegetables; low intakes of high fiber breakfast cereals and fruit.

**Traditional English:**

Frequent intakes of fried fish and potatoes, meats, savoury pies and cruciferous vegetables.

**Dieting:**

Frequent intakes of low-fat dairy products, low-sugar soda; low intake of butter and sweet baked products.

**Low meat:**

Frequent intakes of baked beans, pizza and soy foods; low intakes of meat, other fish and seafood, and poultry.

Host genotypes

Genotyping was conducted with a combination of Illumina arrays (HumanHap300, and HumanHap610Q) [12, 13]. The Illuminus calling algorithm [14] was used to assign genotypes. No calls were assigned if an individual’s most likely genotype was called with less than a posterior probability threshold of 0.95. Validation of pooling was achieved via a visual inspection of 100 random.

Stringent quality control (QC) measures were performed on both arrays prior the imputation stage. We applied two layers of exclusions. The sample exclusion criteria were: (i) sample call rate <98%, (ii) heterozygosity across all SNPs ≥2 standard deviation (SD) from the sample mean; (iii) evidence of non-European ancestry as assessed by principle component analysis comparison with HapMap3 populations; (iv) observed pairwise identity by descent (IBD) probabilities suggestive of sample identity errors; (v) misclassified monozygotic and dizygotic twins were corrected based on IBD probabilities. The exclusion criteria for SNPs were: (i) Hardy-Weinberg equilibrium (HWE) P-value < 10^–6^, assessed in a set of unrelated samples; (ii) minor allele frequency (MAF) <1%, assessed in a set of unrelated samples; (iii) SNP call rate <97% (SNPs with MAF ≥5%) or <99% (for 1%≤ MAF <5%).

After the QC stage the haplotype were estimated from the genotypes (aka phasing) using SHAPEIT v2 [15].

Finally the imputation was performed using IMPUTE v2 software package[15]. The imputation was carried out using 1,000 Genomes haplotypes (SHAPEIT2) -- Phase I integrated variant set release (v3) (September 2013) as reference panel (https://mathgen.stats.ox.ac.uk/impute/data_download_1000G_phase1_integrated_SHAPEIT2.html).

After imputation, regions of 25kb were selected around each lead SNP in Locke et al

[16], and 8,876 SNPs passed further QC (call rate ≥95%, MAF ≥0.05, info > 0.4) and were used for subsequent analysis.

Methylation

DNA methylation profiles were obtained in 542 female Caucasian twins using the Infinium HumanMethylation450 BeadChip assay (Illumina 450k). The DNA methylation Illumina 450k dataset was obtained from adipose tissue biopsies in the twins, as previously described[17] (ArrayExpress E-MTAB-1866). In brief, 8 mm punch biopsies were taken from a relatively photo-protected area adjacent and inferior to the umbilicus. Subcutaneous adipose tissue was carefully dissected from each biopsy, divided into several pieces, and stored in liquid nitrogen until analysis. The beta values on signal intensities for the methylated and unmethylated states were calculated with R. Beta values are the ratio of the normalized intensity of the methylated bead type to the combined normalized locus intensity, and they range from 0 (hypomethylated) to 1 (hypermethylated). DNA methylation levels were first normalized using BMIQ[18], this method transforms type II probes to fit the distribution of type I probes. After categorizing both types of probes into methylated, hemi-methylated and unmethylated, type I probes are used as a base for fitting type II probes into quantiles using inverse of the cumulative beta distributions in each category. The data were visually inspected for outliers. Principal-component analysis (PCA) of the beta values was then performed and the first five principal components were tested for association with potential covariates, including technical (palte, position on plate, batch, bisulphite-sequencing (BS) conversion efficiency, and BS conversion concentration) and biological (age, smoking, alcohol consumption) factors. Beta values on each probe were normalized to N(0,1) then fitted in linear mixed effect models (adjusting with covariates including age, smoking, alcohol, zygosity, family, plate, bisulphite-sequencing (BS) conversion efficiency, and BS conversion concentration). The methylation residuals from this model were then used in the methylation QTL analysis. The methylation QTL analysis was performed using Matrix eQTL[19]. We ran an additive genetic model in matrix eQTL, that is:

Methylation = α + γ (genotype x additive)

where methylation is the normalized value, alpha(α) is intercept and gamma(γ) is the additive genetic effect. We specifically tested for the significance of γ and reporting the t-statistic from the association analysis. The analysis considered genetic association under the additive model between genetic variants at rs74331972, rs1433723, rs2480677 and 467,928 DNA methylation probes that passed quality control.

Supplementary References

1. Jackson MA, Goodrich JK, Maxan M-E, Freedberg DE, Abrams JA, Poole AC, Sutter JL, Welter D, Ley RE, Bell JT, et al: **Proton pump inhibitors alter the composition of the gut microbiota.** *Gut* 2015.

2. Goodrich JK, Waters JL, Poole AC, Sutter JL, Koren O, Blekhman R, Beaumont M, Van Treuren W, Knight R, Bell JT, et al: **Human Genetics Shape the Gut Microbiome.** *Cell* 2014, **159:**789-799.

3. Caporaso JG, Lauber CL, Costello EK, Berg-Lyons D, Gonzalez A, Stombaugh J, Knights D, Gajer P, Ravel J, Fierer N, et al: **Moving pictures of the human microbiome.** *Genome Biology* 2011, **12**.

4. Gilbert JA, Jansson JK, Knight R: **The Earth Microbiome project: successes and aspirations.** *Bmc Biology* 2014, **12**.

5. Caporaso JG, Lauber CL, Walters WA, Berg-Lyons D, Huntley J, Fierer N, Owens SM, Betley J, Fraser L, Bauer M, et al: **Ultra-high-throughput microbial community analysis on the Illumina HiSeq and MiSeq platforms.** *Isme Journal* 2012, **6:**1621-1624.

6. Walters W, Hyde ER, Berg-Lyons D, Ackermann G, Humphrey G, Parada A, Gilbert JA, Jansson JK, Caporaso JG, Fuhrman JA, et al: **Improved Bacterial 16S rRNA Gene (V4 and V4-5) and Fungal Internal Transcribed Spacer Marker Gene Primers for Microbial Community Surveys.** *mSystems* 2015, **1**.

7. Falony G, Joossens M, Vieira-Silva S, Wang J, Darzi Y, Faust K, Kurilshikov A, Bonder MJ, Valles-Colomer M, Vandeputte D, et al: **Population-level analysis of gut microbiome variation.** *Science* 2016, **352:**560-564.

8. Teucher B, Skinner J, Skidmore PML, Cassidy A, Fairweather-Tait SJ, Hooper L, Roe MA, Foxall R, Oyston SL, Cherkas LF, et al: **Dietary patterns and heritability of food choice in a UK female twin cohort.** *Twin Research and Human Genetics* 2007, **10:**734-748.

9. Bingham SA, Welch AA, McTaggart A, Mulligan AA, Runswick SA, Luben R, Oakes S, Khaw KT, Wareham N, Day NE: **Nutritional methods in the European prospective investigation of cancer in Norfolk.** *Public Health Nutrition* 2001, **4:**847-858.

10. Frankenfield DC, Muth ER, Rowe WA: **The Harris-Benedict studies of human basal metabolism: History and limitations.** *Journal of the American Dietetic Association* 1998, **98:**439-445.

11. Willett W, Stampfer MJ: **TOTAL ENERGY-INTAKE - IMPLICATIONS FOR EPIDEMIOLOGIC ANALYSES.** *American Journal of Epidemiology* 1986, **124:**17-27.

12. Richards JB, Rivadeneira F, Inouye M, Pastinen TM, Soranzo N, Wilson SG, Andrew T, Falchi M, Gwilliam R, Ahmadi KR, et al: **Bone mineral density, osteoporosis, and osteoporotic fractures: a genome-wide association study.** *Lancet* 2008, **371:**1505-1512.

13. Soranzo N, Rivadeneira F, Chinappen-Horsley U, Malkina I, Richards JB, Hammond N, Stolk L, Nica A, Inouye M, Hofman A, et al: **Meta-analysis of genome-wide scans for human adult stature identifies novel Loci and associations with measures of skeletal frame size.** *PLoS Genet* 2009, **5:**e1000445.

14. Teo YY, Inouye M, Small KS, Gwilliam R, Deloukas P, Kwiatkowski DP, Clark TG: **A genotype calling algorithm for the Illumina BeadArray platform.** *Bioinformatics* 2007, **23:**2741-2746.

15. Howie BN, Donnelly P, Marchini J: **A flexible and accurate genotype imputation method for the next generation of genome-wide association studies.** *PLoS Genet* 2009, **5:**e1000529.

16. Locke AE, Kahali B, Berndt SI, Justice AE, Pers TH, Day FR, Powell C, Vedantam S, Buchkovich ML, Yang J, et al: **Genetic studies of body mass index yield new insights for obesity biology.** *Nature* 2015, **518:**197-206.

17. Grundberg E, Meduri E, Sandling JK, Hedman AK, Keildson S, Buil A, Busche S, Yuan W, Nisbet J, Sekowska M, et al: **Global Analysis of DNA Methylation Variation in Adipose Tissue from Twins Reveals Links to Disease-Associated Variants in Distal Regulatory Elements.** *American Journal of Human Genetics* 2013, **93:**876-890.

18. Teschendorff AE, Marabita F, Lechner M, Bartlett T, Tegner J, Gomez-Cabrero D, Beck S: **A beta-mixture quantile normalization method for correcting probe design bias in Illumina Infinium 450 k DNA methylation data.** *Bioinformatics* 2013, **29:**189-196.

19. Shabalin AA: **Matrix eQTL: ultra fast eQTL analysis via large matrix operations.** *Bioinformatics* 2012, **28:**1353-1358.
