# Supplementary figures and images for: Heritable components of the human fecal microbiome are associated with visceral fat
Source: Genome Biol. 2016 Sep 26;17:189. doi: 10.1186/s13059-016-1052-7 (PMC5036307; doi:10.1186/s13059-016-1052-7)

**A.**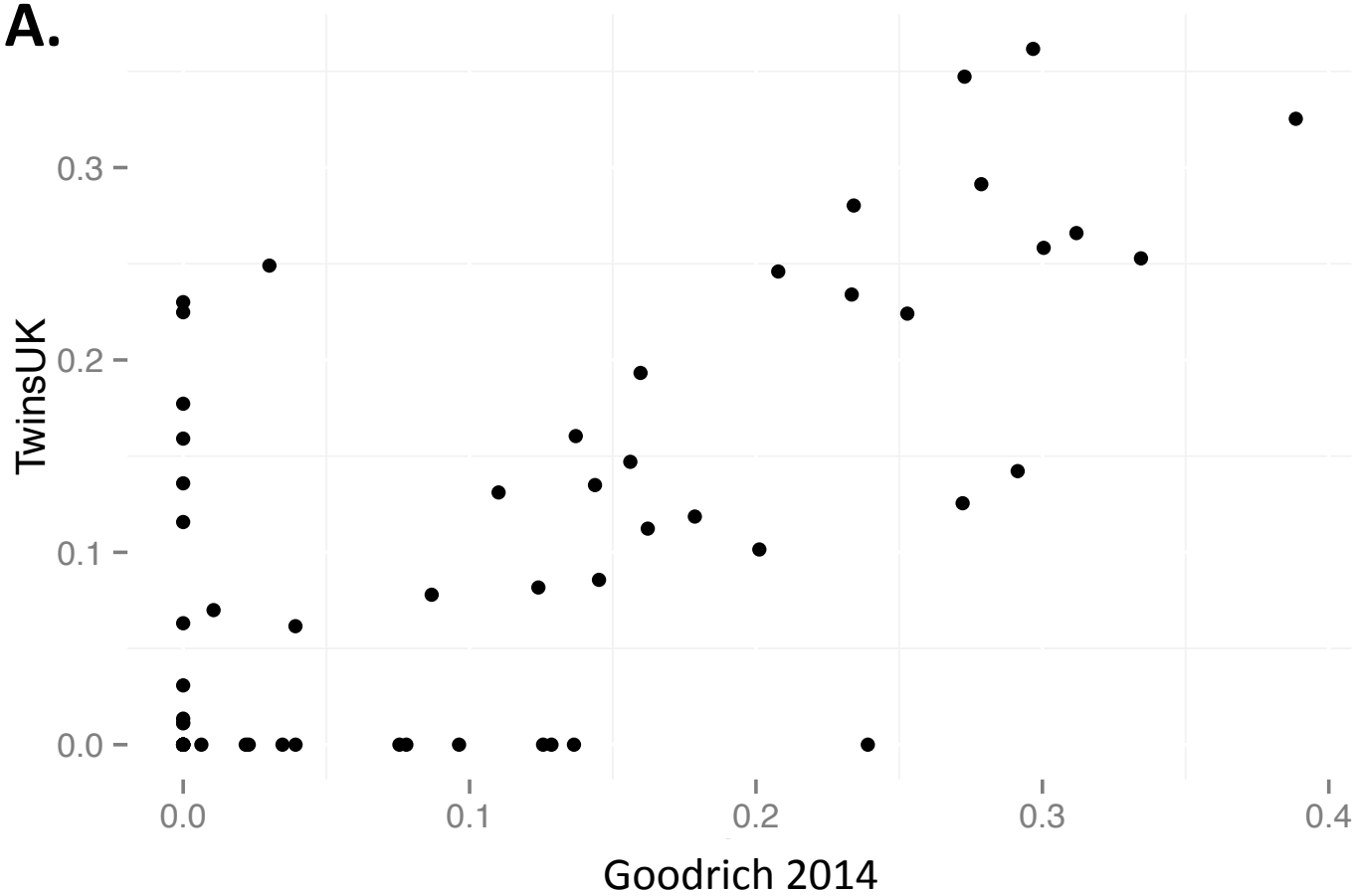**B.**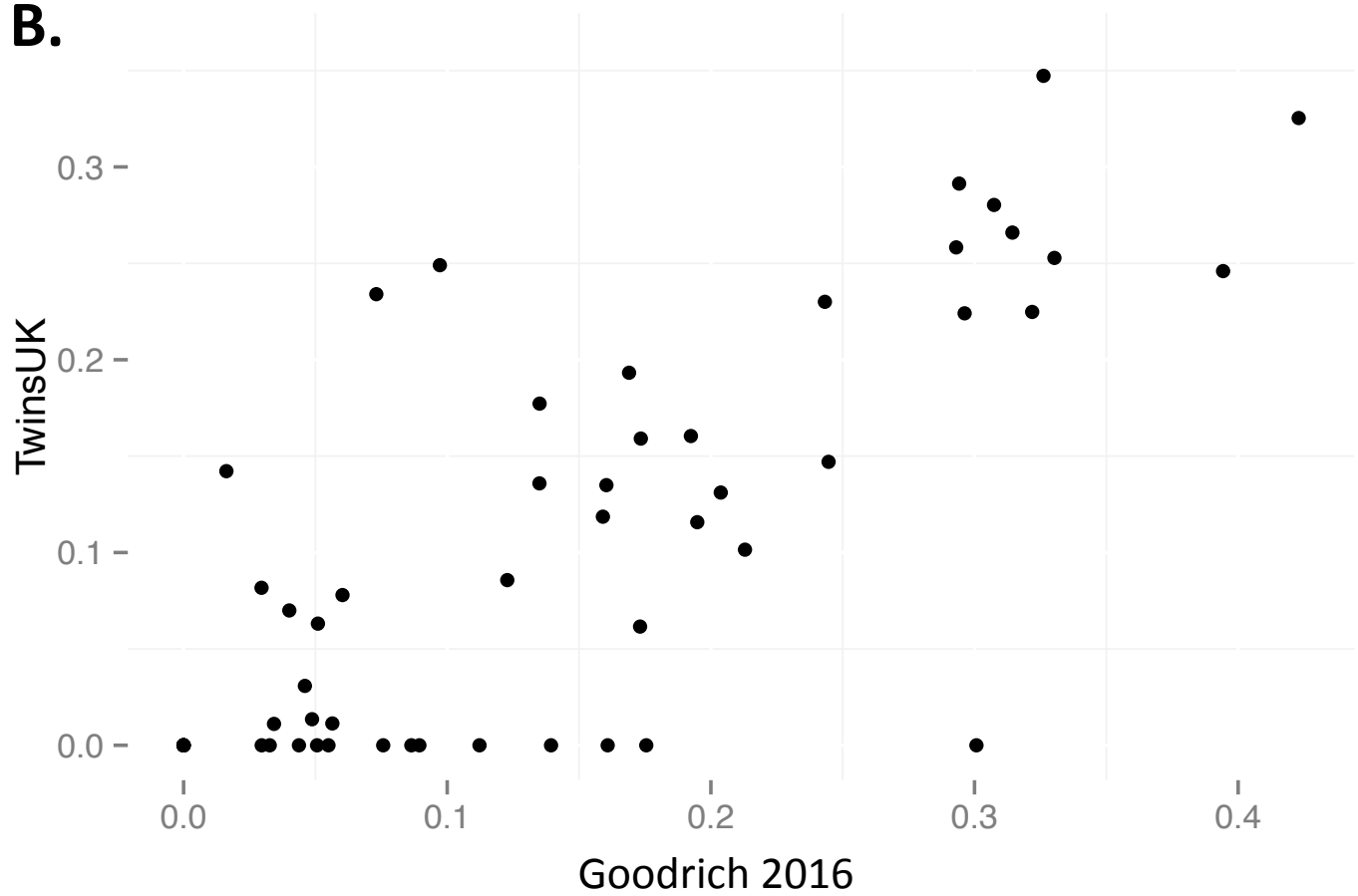

Supplement: Additional file 3: — Genus-level heritability estimates between the TUK discovery dataset and Goodrich. A) Scatterplot showing the genus-level heritability between TUK-D and Goodrich et al. [31] (r 2 = 0.67). B) Scatterplot showing the genus-level heritability between TUK-D and Goodrich et al. [33] (r 2 = 0.76). (PDF 43 kb) [file 13059_2016_1052_MOESM3_ESM.pdf]

Visceral Fat Mass

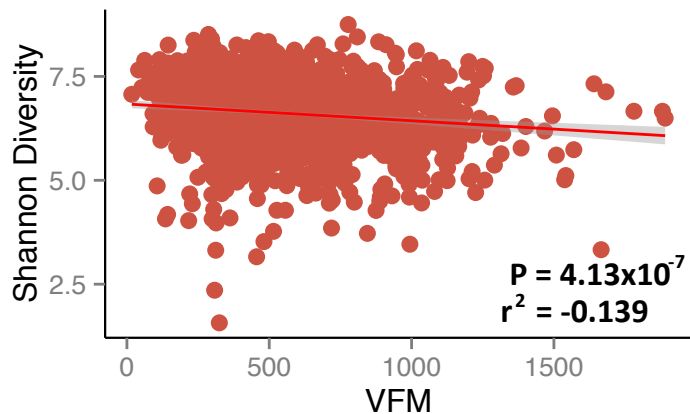

BMI

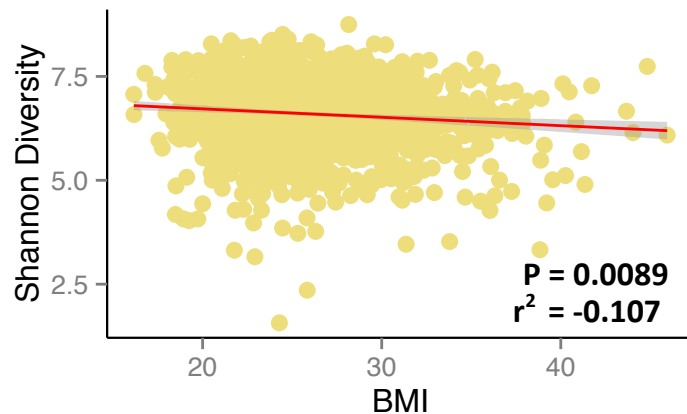

Subcutaneous Fat Mass

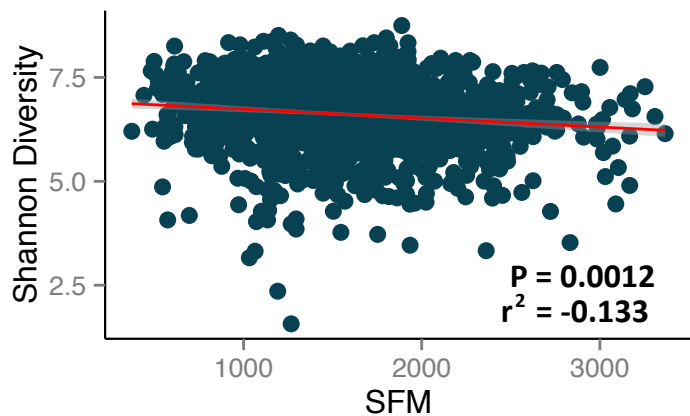

% Trunk Fat

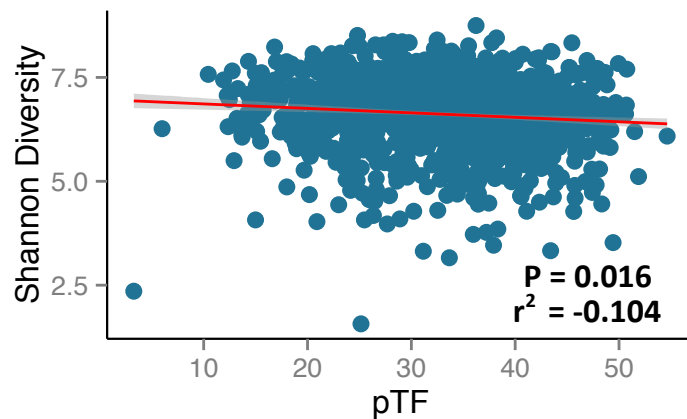

Android:Gynoid Ratio

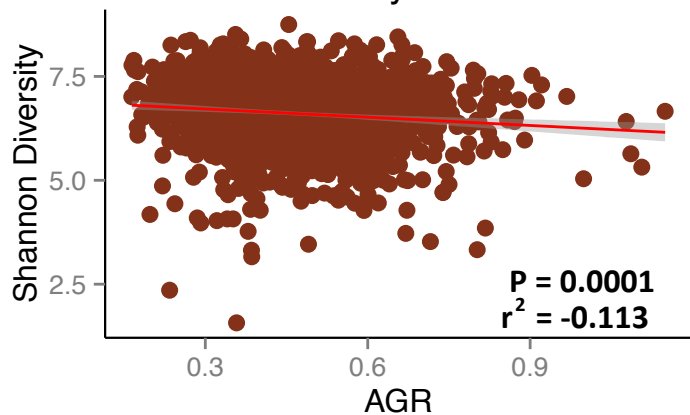

Waist:Hip Ratio

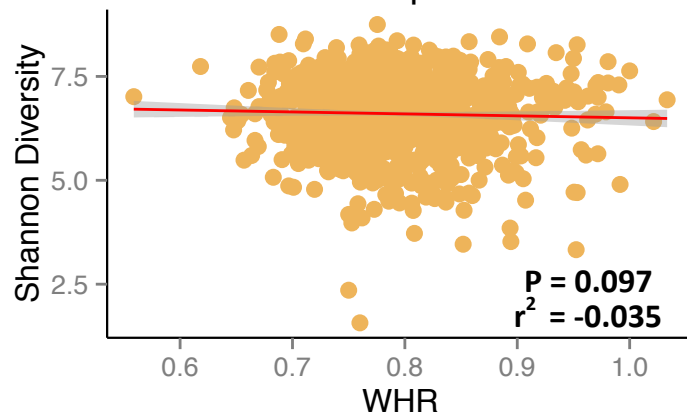

Supplement: Additional file 4: — Comparison of alpha diversity and human adiposity phenotypes. Alpha diversity is measured using the Shannon metric. For each adiposity phenotype considered in this study we present the r 2 and strength of association, and a trend line is shown in red. (PDF 693 kb) [file 13059_2016_1052_MOESM4_ESM.pdf]

**A.**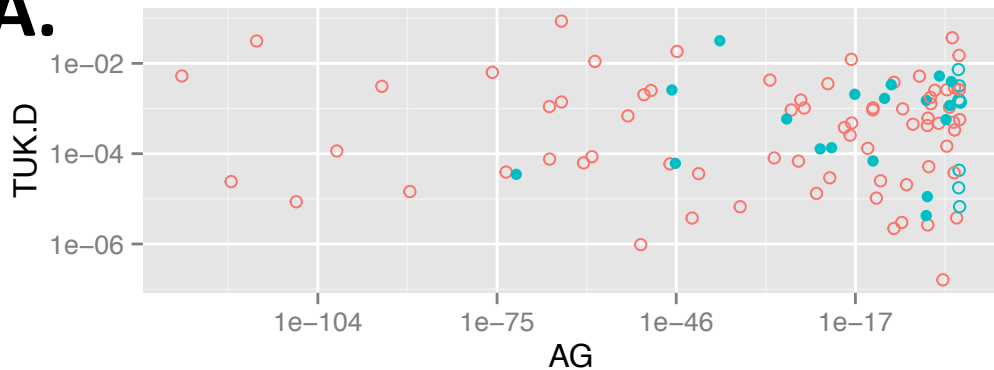**B.**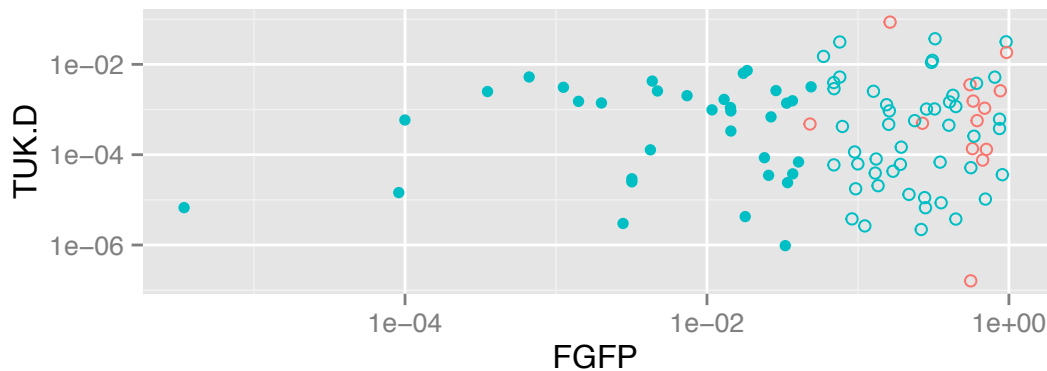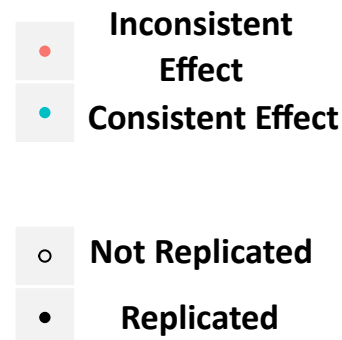**C.**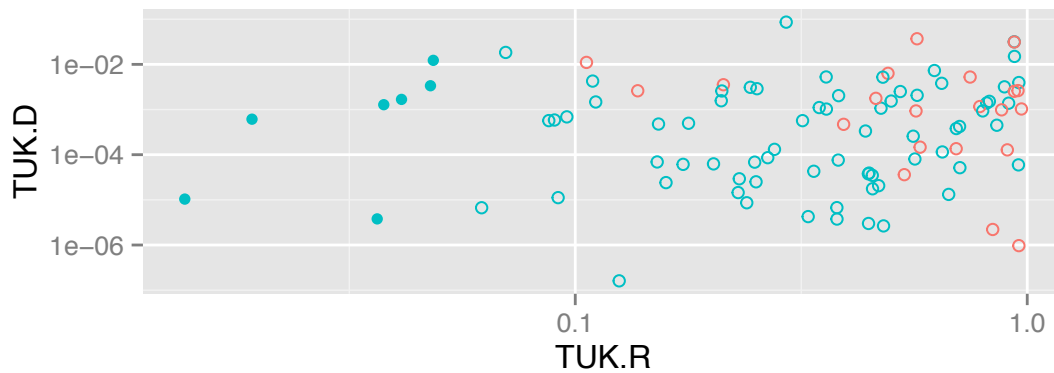

Supplement: Additional file 5: — Concordance of OTU-BMI association results at the 97 OTUs between TwinsUK discovery sample and the three independent replication cohorts. Points marked in red show no consistent effect between the studies, while blue denotes consistent direction of association. Blue points that are empty circles are not significant, while blue filled circles indicate nominally significant results. A) Scatterplot between TwinsUK discovery sample (TUK-D) and American Gut (AG). B) Scatterplot between TUK-D and FGFP. C) Scatterplot between TUK-D and TwinsUK replication sample (TUK-R). (PDF 146 kb) [file 13059_2016_1052_MOESM5_ESM.pdf]
